# Neurophysiological Changes in the First Year After Cell Transplantation in Sub-acute Complete Paraplegia

**DOI:** 10.3389/fneur.2020.514181

**Published:** 2021-01-18

**Authors:** Andrea J. Santamaria, Francisco D. Benavides, Pedro M. Saraiva, Kimberly D. Anderson, Aisha Khan, Allan D. Levi, W. Dalton Dietrich, James D. Guest

**Affiliations:** ^1^The Miami Project to Cure Paralysis, Miller School of Medicine, The University of Miami, Miami, FL, United States; ^2^The Department of Neurological Surgery, Miller School of Medicine, The University of Miami, Miami, FL, United States; ^3^Miller School of Medicine, The Interdisciplinary Stem Cell Institute, The University of Miami, Miami, FL, United States

**Keywords:** spinal cord injury, Schwann cell (SC), transplantation, neurophysiology, level of injury

## Abstract

Neurophysiological testing can provide quantitative information about motor, sensory, and autonomic system connectivity following spinal cord injury (SCI). The clinical examination may be insufficiently sensitive and specific to reveal evolving changes in neural circuits after severe injury. Neurophysiologic data may provide otherwise imperceptible circuit information that has rarely been acquired in biologics clinical trials in SCI. We reported a Phase 1 study of autologous purified Schwann cell suspension transplantation into the injury epicenter of participants with complete subacute thoracic SCI, observing no clinical improvements. Here, we report longitudinal electrophysiological assessments conducted during the trial. Six participants underwent neurophysiology screening pre-transplantation with three post-transplantation neurophysiological assessments, focused on the thoracoabdominal region and lower limbs, including MEPs, SSEPs, voluntarily triggered EMG, and changes in GSR. We found several notable signals not detectable by clinical exam. In all six participants, thoracoabdominal motor connectivity was detected below the clinically assigned neurological level defined by sensory preservation. Additionally, small voluntary activations of leg and foot muscles or positive lower extremity MEPs were detected in all participants. Voluntary EMG was most sensitive to detect leg motor function. The recorded MEP amplitudes and latencies indicated a more caudal thoracic level above which amplitude recovery over time was observed. In contrast, further below, amplitudes showed less improvement, and latencies were increased. Intercostal spasms observed with EMG may also indicate this thoracic “motor level.” Galvanic skin testing revealed autonomic dysfunction in the hands above the injury levels. As an open-label study, we can establish no clear link between these observations and cell transplantation. This neurophysiological characterization may be of value to detect therapeutic effects in future controlled studies.

## Introduction

In early Phase clinical trials of biological therapeutics for SCI, the emphasis is on safety; for thoracic injuries, this is usually considered to be the maintenance of a stable neurological level of injury (NLI). However, the thoracic NLI is based on a sensory exam with no motor component (1). Despite the primary outcome of safety, efficacy signals are sought to inform future product development. Changes in neural connectivity can provide an important “biomarker” when there is a lack of clinically apparent therapeutic effects. Clinical trials have limitations on costs, the available time for study assessments, and the acceptable research burden to participants (2). Drug and biologics development is costly (3), and a lack of an apparent signal of an effect may cause a program to be terminated (4). Whereas, standard outcome measures (5) may fail to detect changes in circuit connectivity (6), neurophysiologic findings may inform development in a therapeutics program.

In this study, we enrolled six thoracic AIS A complete paraplegics with subacute SCI into a dose-escalation safety study of autologous Schwann cell transplantation (aSC) (7). Their neurological level of injury (NLI) was defined conventionally as the last dermatome with normal sensation. In such an injury cohort, the natural history of clinical recovery is minimal (8), especially for upper to mid-level thoracic injuries (9). The clinical neurological examinations are not optimally designed to detect small amounts of recovery (10, 11) and residual connectivity (12) partly because they use ordinal and not continuous motor and sensory score increments (13, 14). Assessment of motor change in the thoracoabdominal region is especially suboptimal, where few clinical or neurophysiological assessments have been used in biologic therapeutics clinical trials (15–17). Pre- and post-transplant electrophysiologic evaluations here explored for the presence of detectable transmission across the sensory-defined NLI, with the null hypothesis that no change would occur from the pre-transplant baseline exam through the 12-month follow-up period.

Neurophysiologic assessments use reproducible stimuli to generate quantitative interval or ratio data (18, 19). Motor and sensory evoked potentials (EPs) and EMG can detect and measure evolving connectivity after SCI (19–22). The accuracy of some prognoses is increased when the clinical exam is evaluated in conjunction with neurophysiology (21, 23) and if EPs are detected in the early post-SCI phases, neurological recovery is more likely (24, 25). Neurophysiologic classification can characterize heterogeneous SCI patterns individually (26) to enable stratified enrollment to clinical trials (27–29). The National Institute of Neurological Disorders and Stroke includes neurophysiologic outcomes as a common data element for SCI (30). A few SCI studies have sought to correlate the clinical neurological exam with EPs (18, 31, 32), but few SCI therapeutics trials have included longitudinal electrophysiology in the participant selection and outcome evaluations (7, 33–37). Motor control can be assessed by testing the ability to initiate and stop EMG activity in a muscle. Although published techniques are available (38), mapping of residual motor control using voluntary EMG signals detectable below the injury level has rarely been reported in a therapeutics clinical trial (36). Studies including intercostal (IC) and abdominal wall EP testing are rare.

For the direct intraparenchymal cell transplant approach used in our clinical trial, safety was a concern. We used detailed EPs during pre-clinical porcine SCI studies (39) to assess the clinical and neurophysiologic effects of increasing injection volumes of aSC into the spinal cord during apneic anesthesia (7). In the porcine model, the maximum tolerated dose of transplanted cells exceeded the spinal cord intraparenchymal pressure tolerance causing MRI-detectable injury correlated with resultant loss of EPs (40) setting a limit for the clinical study. The initial purpose of the EPs was to further define injury completeness (undetectable SSEPs and MEPs across the injury level) within 2–4 days before the *subacute* transplant surgery (40 ± 12.3 days after SCI) (7). We regarded an AIS A exam combined with absent EPs in the context of upper thoracic injury as the most severe injury that could be recruited other than complete spinal cord transection. In the course of these studies, we came to understand there is a rostral-caudal difference between the sensory-defined NLI and the lowest spinal cord levels where residual motor function can be detected. It is thus important to distinguish between the MRI visible maximal injury region, the NLI, and the lowest “level” at which motor activation of the intercostal (IC) muscles can be detected. To make this clear, we introduce the convention to define the intercostal motor level according to the associated intercostal nerve because it is the final conduit for motor activation. Thus, where appropriate, we refer to IC 6 instead of T6.

## Materials and Methods

The six enrolled male participants, number/NLI (001-T3, 002-T6, 004-T1, 006-T4, 008-T4, 009-T4) with traumatic thoracic AIS A injuries completed the entire study, although participant 008 missed his 6-month assessment due to hospitalization. Participants ranged in age from 24–41 years at enrollment (7). All participants had undergone early decompressive and stabilization surgery and were treated per standard of care. The inclusion and exclusion criteria, experimental design, cell preparation methods, transplant procedures, and clinical outcome assessments, including ISNCSCI, MRI, detailed pain evaluations, spasticity, SCIM, and questionnaires for quality of life, are reported (7). Study procedures were approved by the University of Miami Institutional Review Board. The informed consent process complied with the International Declaration of Helsinki and included two consent stages. The first occurred at enrollment to allow nerve harvest and cell production to proceed, and the second after successful autologous cell culture expansion. Following the second consent, the baseline screening assessments (pre-transplant baseline), including SSEPs and MEPs, were conducted in all participants (see Table 1 for the Trial Sequence). Per GCP guidelines, we created standard operating procedures (SOPS) for the neurophysiology assessments with reference to clinical guidelines (41). A manual of operations and EP testing templates with standardized reporting forms were created, signed, and submitted to the study administrator after each session. To improve workflow efficiency and standardize procedures, we conducted extensive testing in control participants. Six non-disabled volunteers, three men and three women, ranging in age from 22 to 45 years of age, underwent the same assessments. In these controls, we tested for optimal cranial sites to evoke intercostal (IC) and leg MEPs, the effect of reinforcement maneuvers, and obtained normative data. Each control participant had at least three independent testing sessions. To evoke MEPs in leg muscles, two stimulation sites, C1/C2 and Cz, were compared as these consistently provide the largest amplitudes in TA and AH, the muscles most likely to show connectivity. TC MEP intensity was tested from 30 to 100% to construct recruitment curves. C1/C2 and Cz stimulation was compared with and without the Jendrassik maneuver, or a 10% (estimated) maximum voluntary contraction, and the signals obtained using surface and needle electrodes compared. For ICs, the relative amplitudes and latencies of CZ stimulation to the right and left sides were compared to C1, C2, and C3, C4 with and without reinforcement maneuvers. Using CZ, we observed asymmetrical values to the right and left side ICs, and thus elected to use C1 and C2 to improve lateralization in the SCI participants.

**Table 1:** Clinical Trial Flow.

** 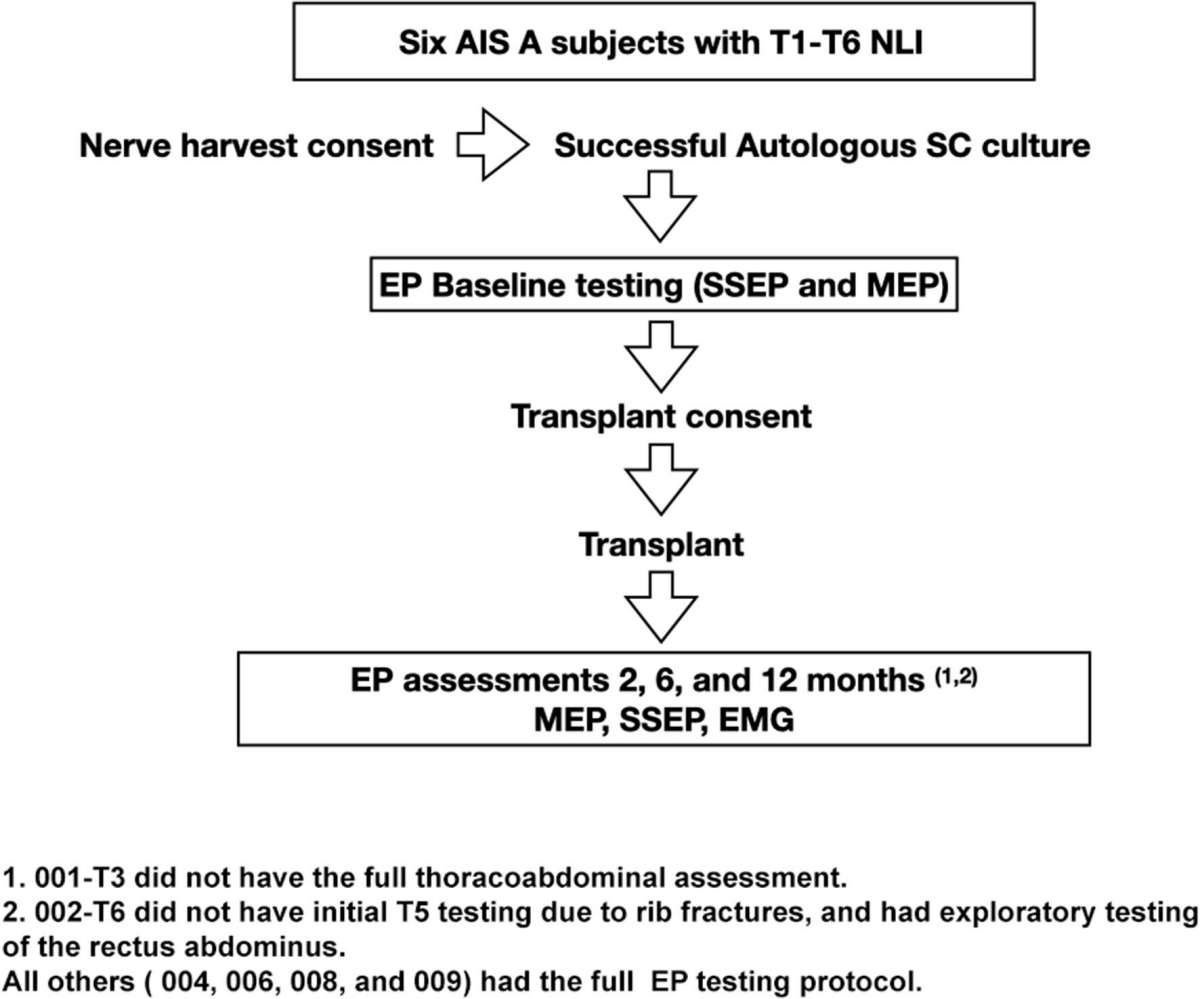 **

The amount of time available for the testing was limited, and we created a time-saving protocol.

The neurophysiological evaluations, including MEPs, SSEPs, voluntarily evoked electromyography (EMG), and galvanic skin responses (GSRs), were conducted within two to four days before transplantation (baseline), and post-transplantation at week 2 and 2, 6, and 12 months. Initial evidence of preserved connectivity detected with MEPs and SSEPs was an *exclusion criterion* for progressing to transplantation surgeries in the initial study.

The senior author conducted a verbal screening assessment before each MEP session to rule out interim changes such as potential seizure activity. Data were collected and analyzed using the FDA-approved Xltek Protektor 32 system running EP Works (Natus Neuro, Ontario, Canada). Steps taken to reduce noise signal included the careful layout of power and signal cables, avoiding crossing or equipment/foot pressure. Impedances were balanced to the extent possible. Sources of capacitive coupling were removed, and signal cables braided to reduce magnetic field interference. A separate common ground electrode was used, and common-mode rejection applied. There were no active IV (intravenous) pumps in the testing rooms. Other identifiable sources of electrical noise were disconnected: beds from their 120 V plugs, IV pumps, room lights, no TV or other equipment, was plugged in, and the neurophysiology equipment power cables were shielded to minimize electrical noise (42). Xltek® Protektor32 IOM data sets were exported for storage, coded, and analyzed by individuals not involved in the participant assessments using Excel, EMGWorks Analysis (Delsys, Natick, MA), and Spike 2 version 9 (Cambridge Electronic Design Ltd). The waveforms were marked for precise latency and amplitude and exported from Xltek EPWorks 6.1 as csv files, so the RMS (root mean square) and MAV (Mean absolute value) could be calculated after the removal of DC bias. In control participants and the first two SCI participants, we explored the best cortical stimulation and recording sites for arm, intercostal (IC), rectus abdominis (RAB), and leg MEPs, including stimulation thresholds in the controls. The number of TcMEP stimuli needed was reduced using arrays to test the upper and lower extremities, chest, and abdomen simultaneously moving the coil on the scalp surface for arm, trunk, and leg stimulation, respectively. The final optimized protocols for IC and abdominal recordings were used throughout the complete timeline of the last four SCI participants (004, 006, 008, 009). The available channels limited the number of IC muscles recorded (IC5-IC8/9). We acquired both chest/abdomen MEPs and voluntary EMG during a deep breath for the comparison of circuit integrity to the magnitude of voluntary activations.

Medications were not discontinued and recorded in the case report form (Table 2). Post-baseline procedures occurred in a well-isolated silent room with participants laying comfortably supine at suitable room temperatures (22–24°C). Participants 002, 004, 008, and 009 had pre-transplant baseline assessments in the wardrooms at Jackson Memorial, where they were admitted for clinical care and rehabilitation. We adapted protocols from previously described MEP and SSEP procedures (12, 41, 43), that followed international guidelines using the 10/20 and 10/10 EEG system for localization (44). Sites on the scalp were defined and marked by measuring the nasion-to-inion, and preauricular-to-preauricular distances, where CZ corresponds to the intersection of the 50% mark of both distances, and the modified combinatorial nomenclature including increments of 10 and 20% of the total distances set the position of the consecutive landmarks for the mid-horizontal and longitudinal lines, respectively (45, 46). For *SSEPs*, skin stimulation sites were exfoliated to obtain measured impedances below five kΩ. Median and tibial nerves were stimulated using bipolar bar electrodes (900-000-202, Neuroline, Ambu, Copenhagen, Denmark) with bilaterally interleaved cathodal monophasic square-wave pulses (0.1 ms pulse duration, 3 Hz, 2X motor threshold of muscle twitch) (41, 47–51). During the SSEP setup, the CMAP for the APB (52) and AH was also acquired for subsequent comparison to the evoked MEPs, if detected, by applying median and tibial nerve stimulation at 20–50 mA and determining the stimulation intensity that lead to a maximal negative peak. SSEP recordings utilized scalp EEG gold cup electrodes and surface monopolar electrodes for skin sites (Neuroline cup electrode and 715-05-K/C, Ambu, Copenhagen, Denmark). The signals were bandpass filtered (3Hz−3kHz) using EPWorks 6.0 (XLTEK, Ontario, Canada). Five hundred to 1,000 sweeps were collected and averaged. Control recording sites for median nerve SSEP stimulation included: Erb's points, the C3 and C6 spinous processes, shoulder and sternum; and cortical C3–C4, Cz, or CPZ-FCZ; and for tibial nerve stimulation: the popliteal fossa, spinous processes of T11 and T12 referenced to the iliac crest, and cortical CP1–CP2, and CPZ–FCZ.

**Table 2:** List of medications received by subjects matched to electrophysiology assessment timepoints.

	**Medication**	**Baseline**	**2 MPT**	**6 MPT**	**12 MPT**
001	Acetaminophen/Oxycodone 5/325 mg/PRN	X	X	X	X
	Alprazolam 0.5 mg/PRN	X	X	X	X
	Baclofen 10 mg/QID	X	X	X	X
	Enoxaparin 30 mg/BID	X			
	Midodrine 5 mg/TID	X	X	X	X
	Zolpidem 10 mg/PRN	X	X	X	X
002	Acetaminophen/Oxycodone 10/325 mg/QID	X	X	X	X
	Baclofen 20 mg/BID		X	X	X
	Enoxaparin 40 mg/QD	X			
	Pregabalin 150 mg/TID		X	X	X
	Solifenacin 10 mg/QD		X	X	X
004	Acetaminophen/Oxycodone 5/325 mg/PRN	X	X	X	
	Baclofen 10 mg/TID		X	X	
	Oxybutynin 5 mg/BID		X	X	
	Pregabalin 75 mg/BID		X	X	X
	Tizanidine 4 mg/TID		X	X	
006	Oxybutynin 5 mg/BID			X	X
008	Acetaminophen/Oxycodone 5/325 mg/QID	X	X	X	X
	Ipratropium/Albuterol 0.5/3 mg/QID		X	X	X
	Aripiprazole 2 mg/QD			X	
	Baclofen 20 mg/BID			X	X
	Clonazepam 0.5 mg/PRN			X	
	Enoxaparin 30 mg/BID	X	X	X	
	Gabapentin 600 mg/TID		X	X	X
	Ibuprofen 800 mg/TID				X
	Oxybutynin 5 mg/BID				X
	Oxycodone 20mg/BID		X	X	
	Sertraline 150 mg/QD		X	X	
009	Acetaminophen/Oxycodone 5/325 mg/PRN	X	X		
	Baclofen 10 mg/QID		X		
	Baclofen 20 mg/QID			X	X
	Diazepam 5 mg/PRN		X	X	X
	Enoxaparin 40 mg/QD	X	X		
	Gabapentin 300 mg/TID		X	X	
	Gabapentin 600 mg/TID				X
	Oxybutynin 5 mg/BID			X	X
	Pregabalin 150 mg/QD		X	X	
	Tramadol 50 mg/BID		X	X	X

*We excluded from the table: antibiotics, vitamins, supplements, antacids, and/or laxatives. Medications were not suspended during follow-up assessments. QD, once a day; BID, twice a day; TID, 3 times a day; QID, 4 times a day; PRN, as needed; MPT, months post-transplant*.

*For MEP* recordings from large muscles, the active, and reference electrodes were placed in the muscle belly 2 cm apart, with the reference distal. Small muscles of the hand and foot were monitored with the active electrode in the muscle belly referenced to the distal tendon insertion. Recording electrode locations were similar to those recommended by SENIAM (53) and a reference textbook (54). MEPs were elicited through a single pulse 100% intensity transcranial magnetic stimulations (TMS) using a double cone coil (110 mm mean diameter) coupled to a Magstim 200^2^ stimulator (Magstim, Whitland, Dyfed, United Kingdom). Stimulation sites were C3/C4, and Cz/C1/C2, for upper and lower extremities, respectively, and Cz/C1/C2 for ICs and AB (55–62). The motor protocol was mainly focused on the detection of axial and lower extremity MEPs in order to use limited time efficiently. Due to the deeper location of the leg M1 cortex, each stimulation utilized 100 percent output power to increase the probability of detecting leg muscle MEPs (63). The initial absence of detectable leg potentials limited the ability to define a hot spot, minimum threshold, an optimal coil orientation for the legs in the participants. At least six repetitions per stimulation point were collected with and without the tested reinforcement maneuvers, including the classic hand grasp-pull (Jendrassik) (64, 65), forceful exhalation (66) and attempted voluntary contractions. Potentials were recorded using paired silver/silver chloride surface electrodes (715-05-K/C, Neuroline, Ambu, Copenhagen, Denmark) from the following muscles for upper extremities: Biceps brachii (BB), extensor carpi radialis (ECR), flexor carpi ulnaris (FCU), abductor pollicis brevis (ABP), and 1st dorsal interosseous (1DI) or abductor digiti minimi (ADM); and lower extremities: tibialis anterior (TA), gastrocnemius (GAS), soleus (SOL), and abductor hallucis (AH). The APB MEP to CMAP ratio was determined from peak-to-peak amplitudes (43) to estimate session to session reproducibility (28). However, this assessment was not possible for the lower extremity due to the initial lack of any detectable MEPs.

As all the participants were clinically complete thoracic paraplegics, we explored methods to record motor signals from the thoracoabdominal area. The ISNCSCI, the leading classification test for SCI used worldwide, does not assess the motor function of the thorax and abdomen; only sensory scores are obtained, and the motor level is considered equivalent to the sensory level (1). Thoracoabdominal control is important to functional recovery; this is a significant assessment gap (67, 68). To approach this question of consistent sensory *and* motor completeness in the thoracoabdominal region, we assessed MEP and EMG recordings from the ICs and RAB muscles of participants 002, 004, 006, 008, and 009. The thoracoabdominal protocol was first modeled in the control participants. Participants 001 and 002 were the first participants enrolled in the study and did not have the finalized thoracoabdominal MEP and EMG examination, including the rectus abdominis at the pre-transplant baseline assessment, although they had all other testings.

We assessed axial muscles, thoracoabdominal [5th to ninth intercostals (IC)], and the rectus abdominis (RAB) (Supplementary Figure 1—human trunk and abdominal recording sites). For IC recordings, we used bipolar bar electrodes with the active site located in each IC space with reference to the sternal border, the midclavicular and anterior axillary lines, and the reference over the immediately superior rib. IC electrode positioning avoided the insertions of the pectoralis minor and was placed anterior to the serratus anterior attachments (69, 70). More rostral ICs had pectoralis muscle overlying them and were not recorded. Needle electrodes were not used for ICs, as some participants initially had multiple painful rib fractures. For the RAB, we did use needle electrodes (12 mm, 27 gauge, Neuroline twisted pair, Ambu, Copenhagen, Denmark) inserted between the midpoint of the xiphoid process and the umbilicus, 2 cm lateral from the midline, with a reference 2 cm above the active needle (71, 72). Hence, RAB recordings correspond to the second right and left abdominis rectus segments. Latencies and peak-to-peak amplitudes were quantified and replicates averaged. All recording electrode localizations were measured from landmarks, detail marked in ink, and photographed for accurate replication in subsequent sessions (73).

**Figure 1:**
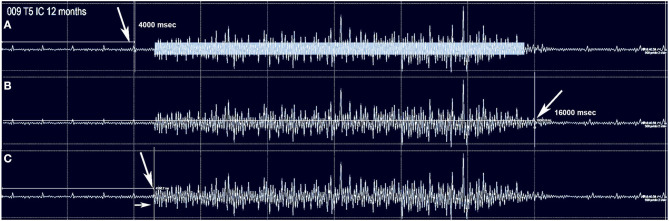
The method used to capture intercostal and abdominus rectus EMG is shown for subject 009-T5. The Jendressick and other reinforcement maneuvers are not used. The subject is trained to initiate a deep breath at the 4 s mark and continue until the 16 s mark if possible. Small EKG spikes are seen in this recording from the T5 intercostal. **(A)** Red arrow indicates the 4 s start marker. The rectangle in **(A)** represents the quantity of EMG signal that is determined from the average EMG during the time-frame, as average amplitude-baseline x time. **(B)** The termination marker is shown, in **(B)**, white arrow. This is important to determine that the signal is not a spasm but is under voluntary control. **(C)** The time gap from the signal to the EMG amplitude increase is calculated, here it is 570 ms.

For voluntarily evoked *EMG*, we followed prior reports such as those used for brain motor control assessment (38, 66). Participants were comfortably supine in a bed with the head elevated 30 degrees after recent bladder catheterization, complete removal of binders, socks, footwear, and inspection for abrasions or skin sores that might trigger spasms. The room was maintained quiet at a comfortable temperature. Relaxation training through controlled breathing was provided before recordings to reduce EMG noise and the frequency of spasms. Participants were familiarized with observing a moving cursor relative to start and stop signals on a large computer screen and trained to initiate and stop the activation of biceps EMG while observing their elicited electrical activity. After completing this training, they were prompted to activate muscles below the injury level in two distinct ways. First, they were asked to contract all their muscles isometrically during the static phase of a breath-hold to see if spasms would emerge. A second round of relaxation ensued, then participants were asked to isolate a contraction to achieve ankle and toe plantar flexion and then great toe dorsiflexion during attempted visualization. We tested both movements with and without reinforcement maneuvers. For the assessment of IC and abdominal voluntary EMG, the participants took a deep breath as the cursor passed the start mark. This breath was held if possible, until reaching the termination marker on the screen, followed by exhalation. The EMG activation observed was recorded within 20-s total windows, with a start and stop signal at four and 16 s, respectively (total 12 s of effort). Signals were amplified and recorded using EP Works without a notch filter (LPF of 10 Hz, HPF of 500 kHz). Attempts were replicated at least six times with 1-min rest between trials. Attempts triggering visually perceptible or suspected spasms were also analyzed to assess for differences from voluntary activity. For analysis, unprocessed EMG signals from EP Works were exported to Microsoft Excel in columns and Delsys EMGWorks Analysis 4.7 as csv text files. Files were imported to Spike2, Version 9 (Cambridge Electronic Design Limited, Cambridge United Kingdom), and digitally low-pass filtered to attempt to filter out EKG artifact. EMG full-wave rectification was performed in EMGWorks, and the signal RMS and the MAV derived. The average peak EMG amplitude was calculated by summing all rectified peaks within the effort window, dividing by the total number, and subtracting the basal noise. An estimate of the EMG output in units of μv-seconds was performed by multiplying the calculated mean amplitude of the best four sequential attempts within the activation window × the duration of the efforts (μV-seconds) (74). Basal noise values were obtained by averaging the amplitude during silent periods of no effort (time 0 to 4 s). EMG bursts were defined as those amplitude changes above the baseline mean (average EMG baseline + ≥ 3 standard deviations) sustained for ≥2,000 ms. A return to the baseline maintained for ≥300 ms was considered the end of the burst (75). The latency of the signal was calculated as the time to generate the first positive burst after the ON command at the 4-s time-point (76). The delay and proportion of continuous positive EMG signals were determined relative to the 12-s volitional attempt window (see Figure 1).

To assess the ability to trigger changes in skin electrical conductance, we recorded the galvanic skin response (GSR) as the change in potential above baseline evoked by a deep breath and exhalation stimuli (77). Signals were collected using an iPhone-based biofeedback electrode system (Esense, Mindfield Biosystems Ltd, Gronau Germany at 10Hz) (78). The GSR is similar to the SSR (sympathetic skin response) (79) but allows the pre-stimulus signal baseline conductance and fluctuation to be recorded before stimulus initiation. The skin was gently cleaned from oils, and two disposable gel electrodes were placed on the glabrous skin of the first and second fingers and toes, respectively. The skin temperature was measured and maintained above 32°C. Participants were trained to relax and breathe without elevating the conductance displayed as a continuous red line on a screen image. With progressive relaxation, it is normal for the current to drop in the upper extremities progressively. A digital stopwatch was used to conduct the test, and participants were instructed to initiate three deep breaths in sequence. The time to peak response and skin conductance changes in μSiemens were recorded (1S = Ω^−1^). Three replicates per limb were obtained, separated by periods of at least 2 min to reduce habituation. We exported the data as .csv files, analyzed in Excel, and plotted as △μS vs. time.

Statistical comparisons. In the control group, we tested for a correlation between thoracoabdominal MEP amplitudes and the average maximum amplitude of the IC and RAB EMG signal during the voluntary breath-hold. The data from at least three sequential replicates were analyzed in XLSTAT 1019.1 using a Pearson correlation test for continuous data and linear regression to determine the *R*^2^-value. The same testing was applied to the MEP/EMG datasets of participants 004, 006, 008, and 009. We also assessed for evidence of a thoracoabdominal neurophysiological transition level to indicate a boundary between greater and lesser amplitudes and latencies for MEPs and EMG signals. The amplitude changes at potential thoracic transitional levels over time were compared using regression analysis to test the hypothesis that positive slopes would be observed at each level in GraphPad Prism 8. A positive slope would indicate progressively improved IC activation over time, as might be expected during recovery from SCI.

The IC and abdominal MEP latencies and amplitudes were analyzed by creating nested tables in GraphPad Prism. Each IC/abdominal level and the three measurement time points were nested. This data was analyzed by one-way ANOVA to compare columns, the homoscedasticity of the data derived, and the dataset means and confidence intervals plotted. Amplitude and latency met the requirement of approximate normality.

## Results

### Participants

By chance, the six participants all had an NLI of between T1–T6. The ISNCSCI sensory levels defining the NLI and the sensory zone of partial preservation (ZPP) at baseline, 6 and 12 months are shown in Supplementary Figure 2. For MEPs, the first several acquired were largest in terms of amplitude whereas, for voluntary EMG, the first 3- 4 attempts were relatively weak and then became stronger. To calculate averages, we used the largest 4–5 sequential traces.

### Controls

We considered the TA and AH to be the leg muscles most likely to be detected (80) in the SCI participants and extensively tested the control participants to optimize our protocol (Supplementary Figure 3). MEP amplitudes from midline Cz stimulation were marginally larger than from C1/C2, and this was consistent for the added effect of the Jendressick maneuver. However, the C1/C2 coil location provided more specific lateralization than Cz. The 10% maximum voluntary contraction was more effective in increasing amplitude than the Jendressick maneuver. Observed amplitudes were consistently larger for the AH than TA. For IC MEPs, Cz stimulation and C1/C2 to the right and left sides were compared with and without the Jendressick maneuver. Stimulus intensity recruitment curves were constructed.

**Figure 2:**
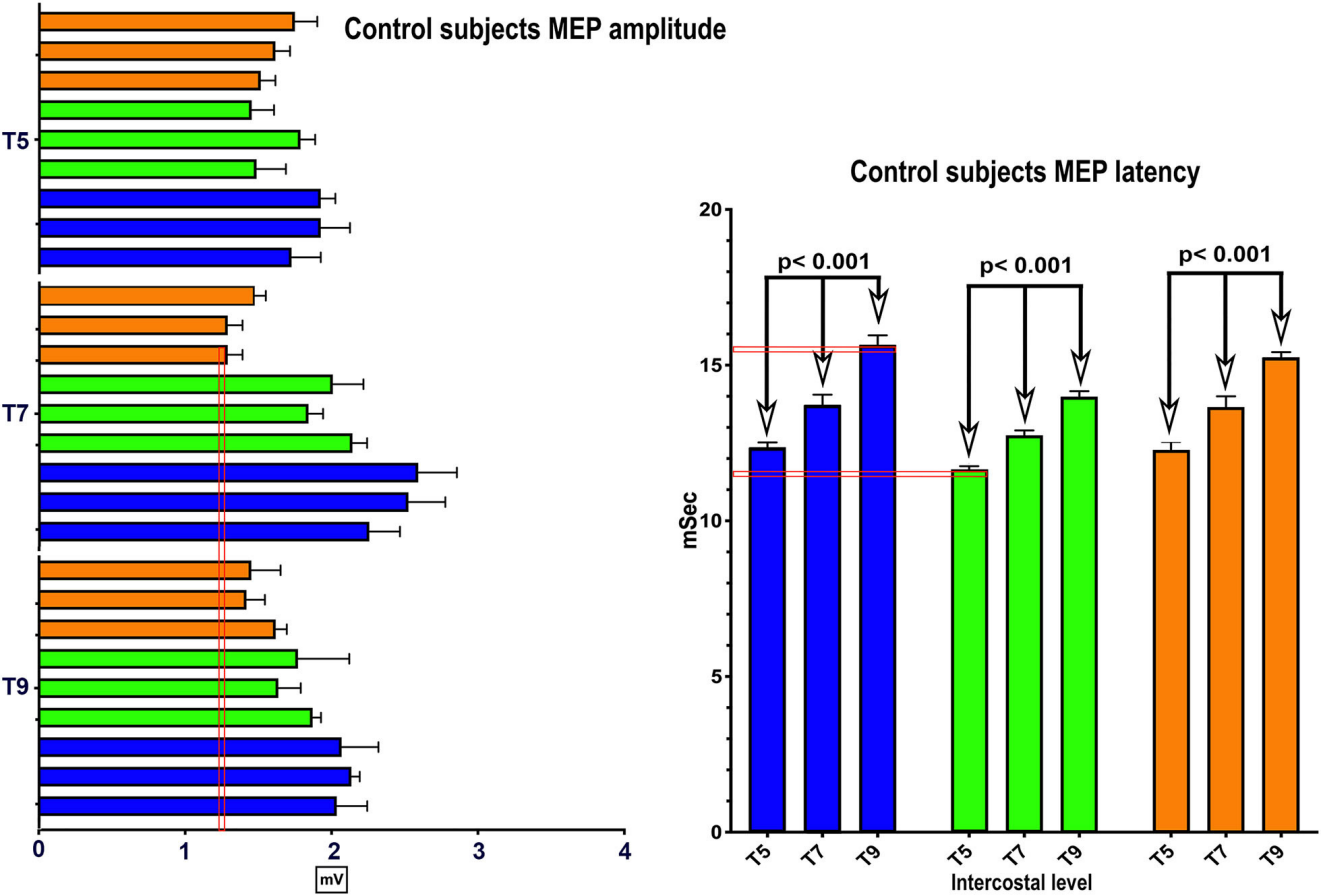
MEP control values. The same methods were used in 3 control subjects to determine the average amplitude and latency of intercostal MEPs using 100 percent coil output. While there were inter-individual differences, the within individual latencies showed little variability. Amplitudes varied generally from 1–2 mV.

No clear trend for MEP amplitude differences per level between IC 5–9 was apparent in the control participants with the smallest MEP being 1.25 mV, but latencies increased at more caudal levels with ICs 5, 7, and 9 each differing statistically from the other (Figure 2), (2-way repeated-measures ANOVA, *P* < 0.0001, Row factor for different IC levels F = 63.85, Geisser-Greenhouse epsilon for sphericity = 0.85). The range of latencies between IC 5 and IC 9 in control participants was 11.4 to 15.5 ms.

**Figure 3:**
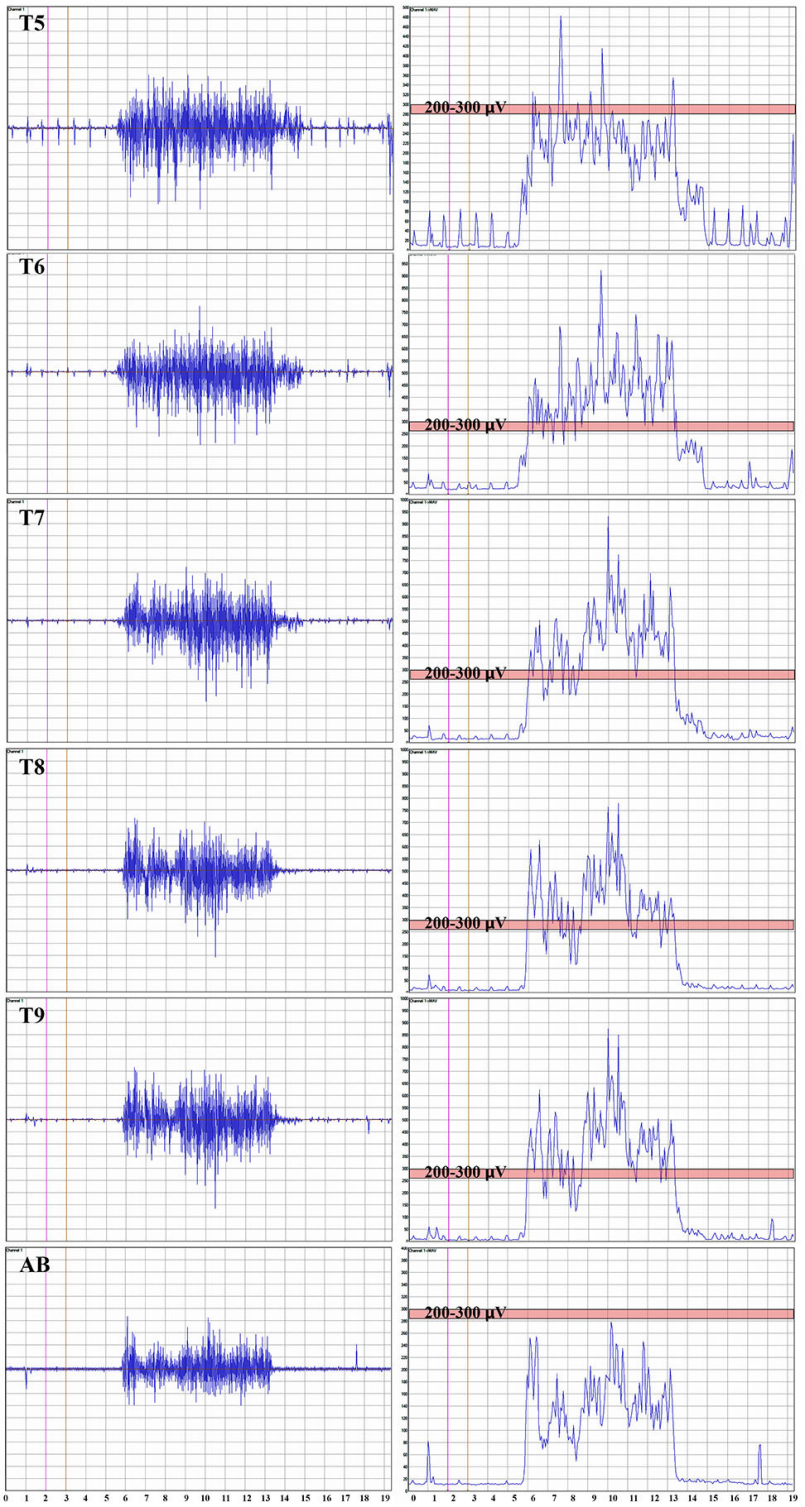
Thoracoabdominal EMG recordings with Mean Absolute value. These recordings are from one female control individual during a timed deep breath hold. The collected surface EMG over the intercostals and the rectus abdominis are shown by level. The baseline noise is small (~15 μV). There is some EKG signal also detected, and in this example, not removed. On the right side of the panel, the signal mean absolute value is shown to provide a visual reference to the signal size. The EKG is even more obvious. A pink bar with its upper margin at 300 μV is shown to provide a reference to the amplitudes between the respective intercostals and rectus abdominis. The T5 to T9 signals are relatively similar with the rectus abdominus being smaller.

Representative average IC EMGs in the breath-hold window from a control participant is shown in Figure 3. The EMG signal is rectified, and the mean absolute value (MAV) is shown for each level from IC 5 to AB. The signal quantity is similar between IC 6-IC 7-IC 8 and IC 9, while the AB is smaller. The average MEP and mean EMG amplitudes per level from IC 5-IC 9 + AB in control participants were correlated, e.g., control participant 2, *R*^2^ = 0.44, *p* = 0.009.

Median and Tibial CMAPs were obtained during the preparation for the SSEPs, and examples are shown for participant 006 at 6 months post-transplant in Supplementary Figure 4. The amplitudes used for calculation were the total peak-to-peak resulting in 8.7mV/14.38mV = 0.61. The overall MEP/CMAP ratio for the APB from control sessions was relatively high at 0.58 +/− 0.19 mV, as has been reported with 100% TMS stimulation (81). Although tibial CMAPs have been reported to be consistent after thoracic SCI (82), the absence of leg MEPs until 6 months post-transplant prevented their use for session-to-session normalization.

### Participant Tolerance

The average neurophysiology session duration was 6 h, including preparation and breaks, except for participant 009, who was studied on two consecutive days after TA and AH MEPs were detected at the 6-month time point. No significant complications were associated with electrophysiology assessments. Some participants reported transient headache after TMS sessions, which resolved spontaneously or after NSAID administration.

### Electrophysiologic Motor Assessment of the Lower Extremities

The EP testing evaluated for evidence of cortical motor-evoked or voluntary activation of muscles of the legs. Several leg muscles were evaluated, but activations were only detected in AH, TA, GAS, and SOL in order of frequency (Table 3, motor summary), Excel trial motor data spreadsheet). For MEPs, signals were most commonly observed in the TA, whereas for voluntary activation, AH was most common. Both increased MEP latencies and delayed activation of voluntary EMG were consistently observed. The amplitudes of most MEP and EMG signals were <100 μV, but increases between 6 and 12 months were observed, and 009 showed the largest values at the 12-month endpoint (260 μV with Jendressick reinforcement).

**Table 3:** Presence or Absence of Motor Signals.

	**Subject**	**001-T3**		**002-T6**		**004-T1**		**006-T4**		**008-T4**		**009-T4**	
	**Side**	**R**	**L**	**R**	**L**	**R**	**L**	**R**	**L**	**R**	**L**	**R**	**L**
B	GAS MEP	0	0	0	0	0	0	0	0	0	0	0	0
2		0	0	0	0	0	0	0	0	0	0	0	0
6		0	0	0	0	0	0	0	0	0	0	0	0
12		0	0	+	0	0	0	0	0	0	0	0	0
B	GAS EMG	0	0	0	0	0	0	0	0	0	0	0	0
2		0	0	0	0	0	0	0	0	0	0	0	0
6		0	0	0	0	0	0	0	0	0	0	0	0
12		0	0	0	0	+	0	+	+	0	0	0	0
B	TA MEP	0	0	0	0	0	0	0	0	0	0	0	0
2		0	0	0	0	0	0	0	0	0	0	0	0
6		+	0	+	0	0	0	0	0	0	0	+	0
12		+	0	+	+	0	0	0	0	0	0	+	+
B	TA EMG	0	0	0	0	0	0	0	0	0	0	0	0
2		0	0	0	0	0	0	0	0	0	0	0	0
6		0	0	0	0	0	0	+	0	0	0	0	0
12		0	0	0	+	+	0	+	+	0	+	+	+
B	SOL MEP	0	0	0	0	0	0	0	0	0	0	0	0
2		0	0	0	0	0	0	0	0	0	0	0	0
6		0	0	0	0	0	0	0	0	0	0	0	0
12		0	0	0	0	0	0	0	0	0	0	0	0
B	SOL EMG	0	0	0	0	0	0	0	0	0	0	0	0
2		0	0	0	0	0	0	0	0	0	0	0	0
6		0	0	0	0	0	0	0	0	0	0	0	0
12		0	0	0	0	+	+	0	0	0	0	+	0
B	AH MEP	0	0	0	0	0	0	0	0	0	0	0	0
2		0	0	0	0	0	0	0	0	0	0	0	0
6		0	0	+	0	0	0	0	0	0	0	+	+
12		0	0	+	+	0	+	0	0	0	0	+	+
B	AH EMG	0	0	0	0	0	0	+	+	0	0	0	0
2		0	0	0	0	0	0	+	+	0	0	+	0
6		0	0	0	0	0	0	+	+	0	0	+	+
12		0	0	0	+	+	+	+	+	+	+	+	+

#### Lower Extremity MEPs

**Figure 4:**
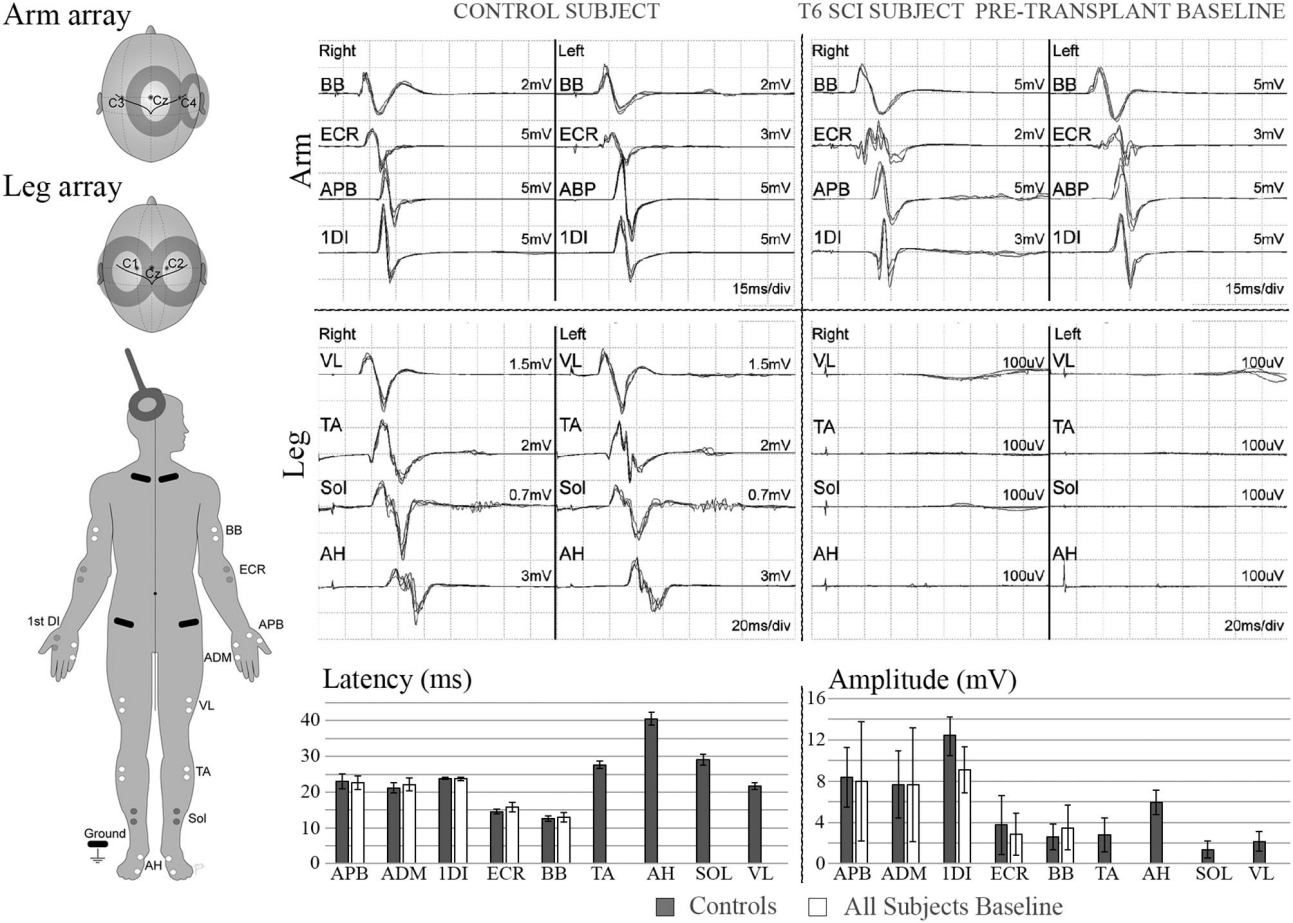
MEPs. Left diagram shows recording and stimulation sites for MEPs. 100% intensity TMS is used to elicit the potentials. Representative initial MEP recordings from a non-injured vs. a T5 SCI subject are shown for several upper and lower extremity muscles. Scale in lower extremities is set to 100 μV to confirm absence of potentials. Lower panel shows latency and amplitude values from control and injured subjects. Control values include recordings from 3 able bodied individuals, bars show average ± SD from 7 MEP trials per muscle. Some of initial variability in the upper extremity is due to the SCI subject with brachial plexus injury.

Representative stimulation and recorded waveforms from participant 002 baseline compared with the non-injured control participants are shown in Figure 4, along with summary pre-transplant MEP baseline data recorded from the six participants and non-injured control individuals. MEP latencies were stable, but amplitudes showed intra and inter-participant variability.

**Figure 5:**
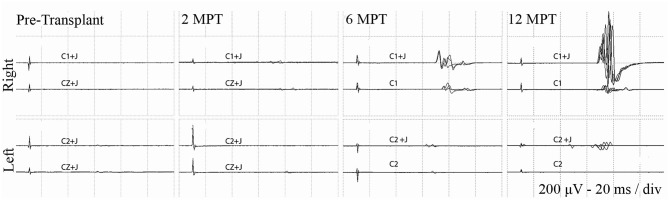
Effect of Jendressick maneuver on MEP amplitude. Serial AH MEPs from subject 009-T5. TMS is delivered at 100% at C1/C2 and CZ. Every time-point of the study is shown for right and left side and for stimulation with and without Jendrassik maneuvers (+J). Positive signals are clearly identified at 6 months. MEP amplitude increases at the 12-month follow up, but remains delayed in latency at ~60 ms.

**Figure 6:**
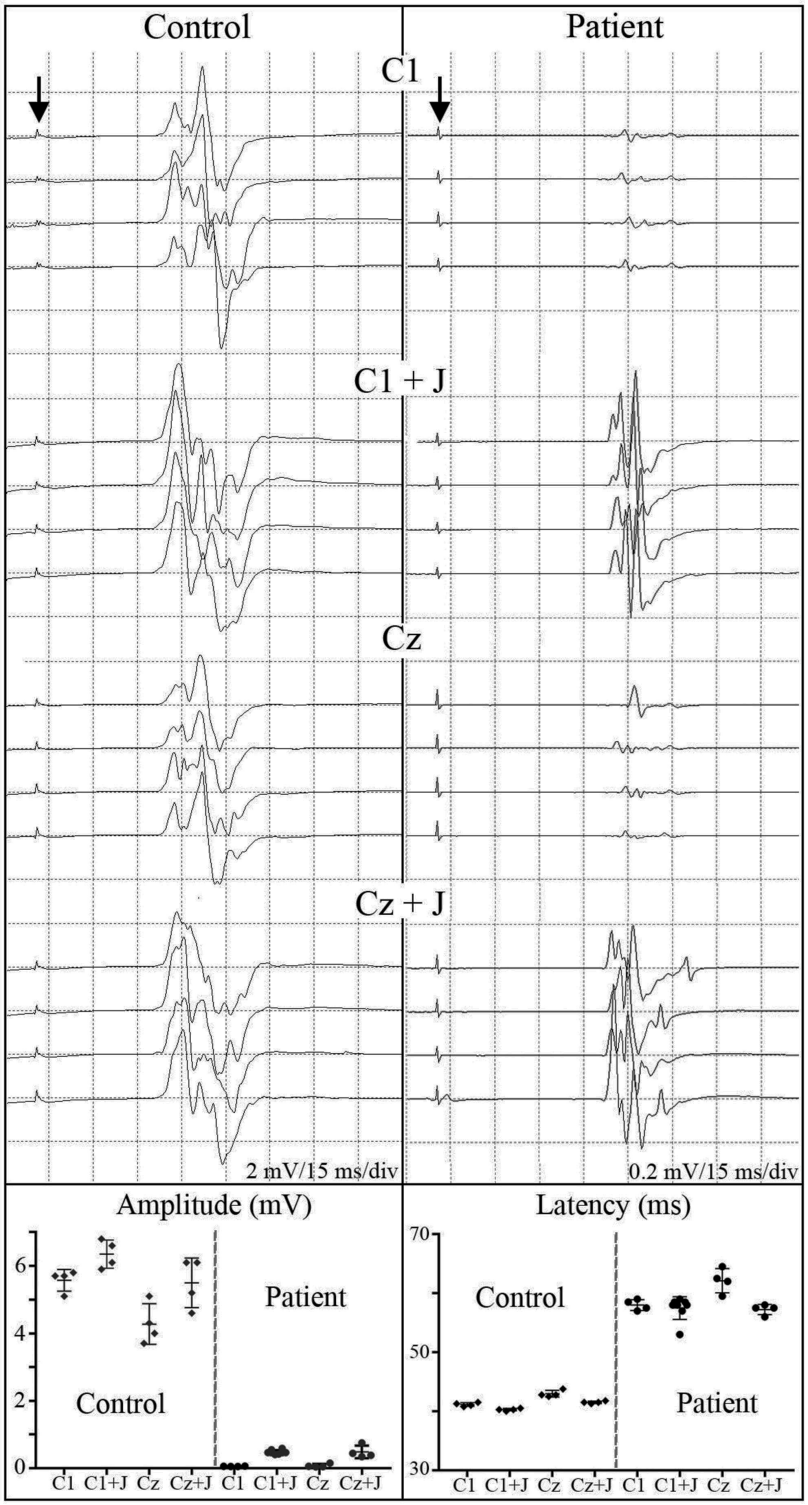
C1 vs. Cz stimulation with and without Jendrassik facilitation influences MEP detection. Individual waveforms recorded in sequence during a continuous session are shown to indicate waveform consistency. A control non-SCI individual compared to a T5 SCI subject at 12-month post-transplantation follow-up. Vertical arrows indicate stimulation artifact. The influence of the C1 vs. Cz position is much less evident than the marked potentiating effect of the Jendressick maneuver. In controls, +J amplitude increase corresponds to 14% for C1 and 28% for Cz, in the SCI subject 879% for C1 and 671% for Cz. Latencies show slight decrease in control of 3% for C1 and 4% for Cz, in SCI subject 3% for C1 and 8% for Cz.

At 2 months post-transplant, lower limb MEPs were absent, but 2 participants (006, 009) had small detectable voluntary EMG activations (Table 3, Motor Summary, Supplementary Figure 5, and complete motor data sheet.xls). At 6 months, MEPs were detected in 3 participants (001, 002, and 009), and voluntary EMG was present in participants 006 and 009. At 12 months, MEPs were detected in 4/6 (001, 002, 004, and 009) and vEMG in 5/6 (002, 004, 006, 008, and 009). Recordings from the AH of participant-009, at baseline, 2, 6, and 12 months are shown in Figure 5. The Jendrassick maneuvers increased the MEP amplitude and moderately decreased latency; without augmentation, it would be easy to miss these MEPs. The TA MEPs at 12MPT as compared to a control non-SCI individual are shown in Figure 6. Note the reproducibility, prolonged latency, potent effect of reinforcement maneuvers, and the small but consistent amplitudes 10x less than the control participant. The latency (55.5–57.5 ms) was more prolonged than in control individuals whose average was ≤40 ms. The amplitudes (<1 mV) were still not associated with detectable movement during the ISNCSCI motor exam (scored 0/5). The tibial nerve CMAP from the AH was recorded using methods described by Kirshblum et al. (83). It was 4.7 ± 0.3 mV. At 12 months, the AH MEP was 0.13 ± 0.3 mV, resulting in an MEP/CMAP ratio of 0.027. Regarding the site of optimal scalp surface stimulation for this participant, the MEP amplitude was slightly larger for C1 vs. Cz stimulation. Besides the +ve MEP findings, this participant converted from AIS A to AIS B based on anal sensation. The conversion was first detected at six MPT and maintained at the 12 MPT follow-up.

#### Lower Extremity Electromyography Recordings

An EMG protocol was used to assess for activation of lower limb muscles in the control group and all six participants. For these recordings, we focused on establishing a quiet EMG baseline, capturing the onset latency of the detected EMG signal measured from a GO command, the ability to stop the contraction effort, the amplitude of the rectified signal, and the percentage of signal maintained through the effort window (Summarized in Table 3 with data in the Excel Spreadsheet). 5/6 participants had some detectable *voluntary* EMG at the study endpoint. The delay in eliciting voluntary leg EMG varied from 0.86 to 3.4 s. Two participants had early detectable voluntary EMG, 006-T4 had small bilateral small signals in the AH at the baseline, and 2-month evaluation. 009-T4 also had voluntary EMG detectable at the 2-month post-transplant time point (Supplementary Figure 5); at 6 months, the EMG was 6x greater in amplitude and filled the time window. The onset delay and fatigue limited the fraction of the time window that was positive. For the legs, the fraction varied from 26 to 80%, while in control participants, the activation delay was negligible. We also observed occasional spasms in EMG recordings in these participants. Spasms differed from voluntary activations with peak amplitudes of several hundred microvolts or into the millivolt range. Usually, spastic activity was visually apparent, and participants could not voluntarily stop the contractions (Supplementary Figure 6).

### IC and Abdominal MEP and EMG Assessments

#### EMG

**Figure 7:**
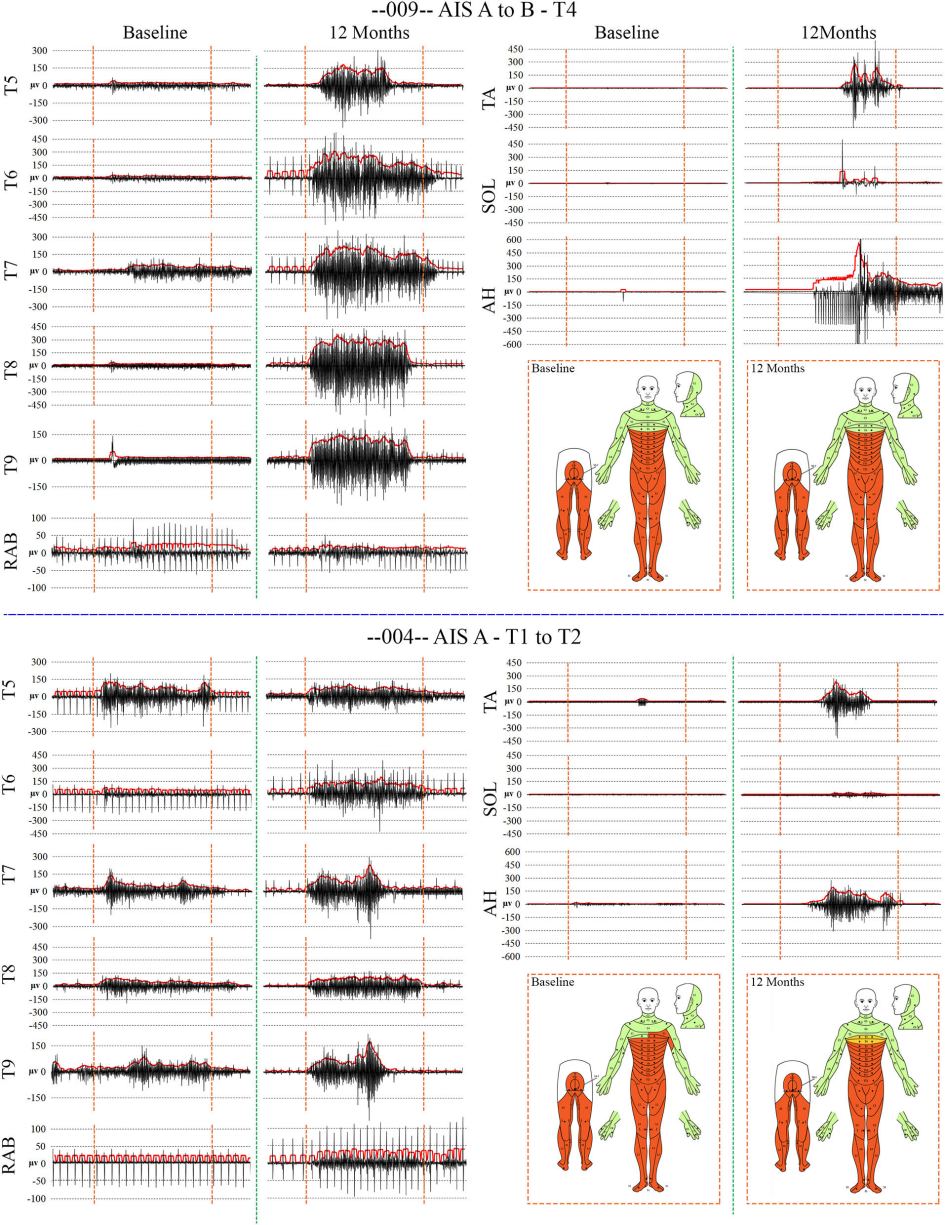
Recordings of voluntary intercostal and leg muscle EMG at baseline and 12 months post-transplantation with comparison to the NLI. Subjects 009 and 004 have sensory exam defined levels of T4 and T1, respectively. The EMG root mean square is shown in red. The deep held breath and the leg EMG are recorded separately. Breath initiated activity improves over time despite no change in the sensory level. Voluntary leg muscle activation was evident in 5/6 subjects in the AH muscle at endpoint. In the 12 month recording show for 009, the muscle activity in TA, SOL and AH is initiated near simultaneously, but the AH activity cannot be voluntarily stopped. We did not consider EMG activation that could not be voluntarily stopped as “+ve” recordings, although it is possible that a voluntary activation could trigger a spasm.

When breathing quietly at rest, there was minimal IC EMG activity in the participants with a timed deep breath, all participants had small but detectable EMG activity recorded from IC segments at baseline with amplitudes that mainly increased at the study endpoint. In Figure 7, baseline vs. 12 MPT EMG activity from a participant with substantially improved IC voluntary EMG changes (009-T4) is shown. Participant 009 showed positive voluntary EMG signals across almost the entire effort window in the ICs with a clear ability to stop upon exhalation. The reduced initial EMG amplitudes may reflect pain, deconditioning, weakness of the chest bellows unit, and reduced axonal transmission. The temporal changes are consistent with observations of previously reported improvements in chest function during evolution from acute to chronic injury (84). With repeated deep breaths, our participants often exhibited a marked reduction in the area of the effort suggestive of fatigue.

We had not anticipated that the rectus abdominis (RAB) muscle could be positive, and thus it was not assessed in participant 001. We fortuitously detected the signal in participant 002 and added it to the assessment protocol using needle electrodes for specificity. RAB MEPs and EMG were negative at baseline in 004 and 008 but positive at other time points. EKG artifact was present in all channels, and a low pass differentiator filter applied in Spike2 to remove it was not satisfactory as substantial signal was lost (Supplementary Figure 7). In another publication in preparation, we have used the Spike2 independent component analysis (ICA) script to extract the EKG artifact from the EMG.

#### MEPs

**Figure 8:**
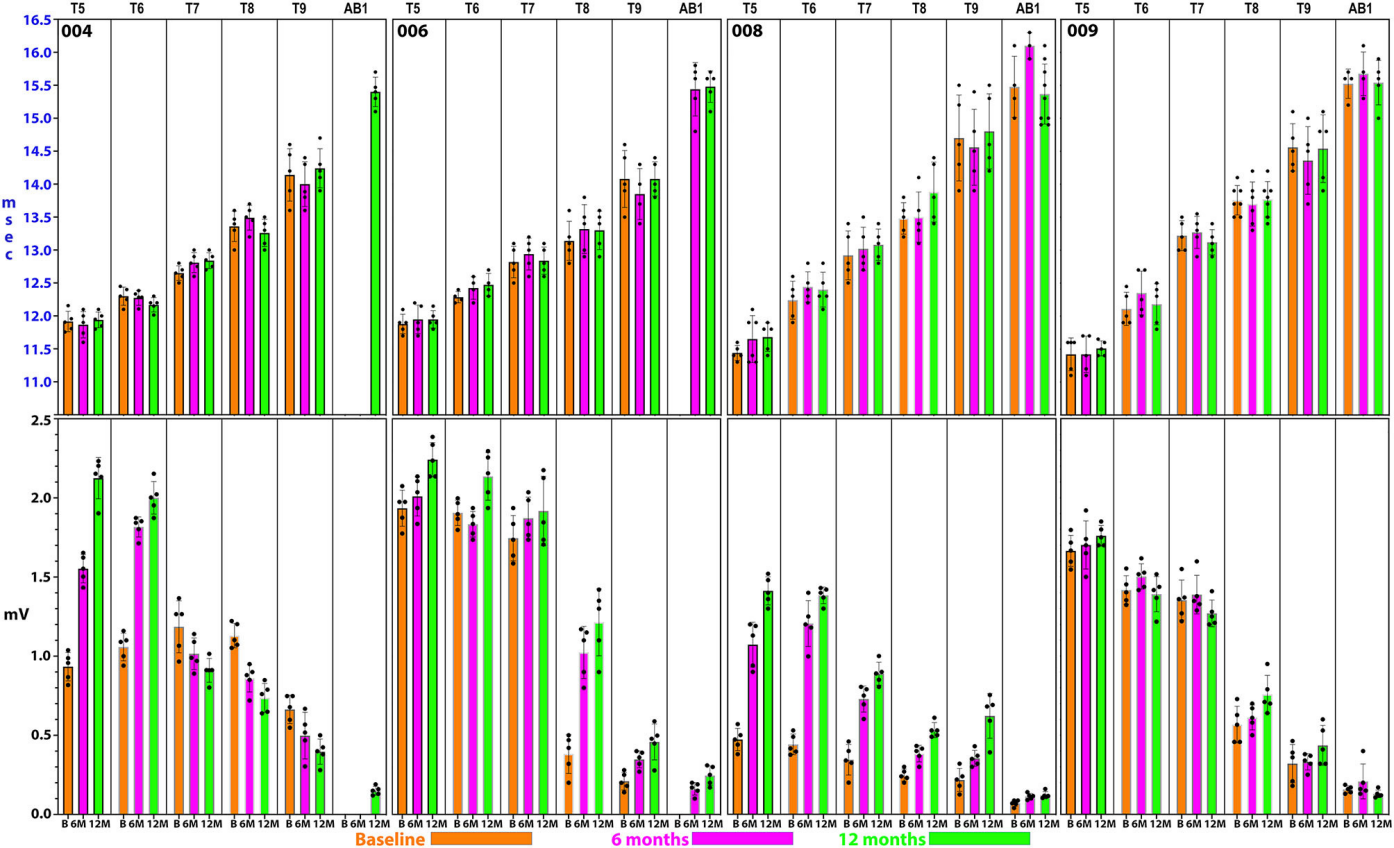
Detailed thoracoabdominal motor evoked potential latency and amplitude. Shown are 5 sequential MEPs recorded per level from a simultaneous montage in subjects 004, 006, 008, and 009 at baseline, 6, and 12 months. The latencies show less variation than the amplitudes with the exception of T9 and the rectus abdominus. 004 and 008 have lower amplitudes at the baseline possibly due to acute injury effects. Later time point amplitudes increase in all except 004, T7 and below. Amplitudes show a trend to cluster in 2 groups, T5–T7, and T8–AB1.

**Figure 9:**
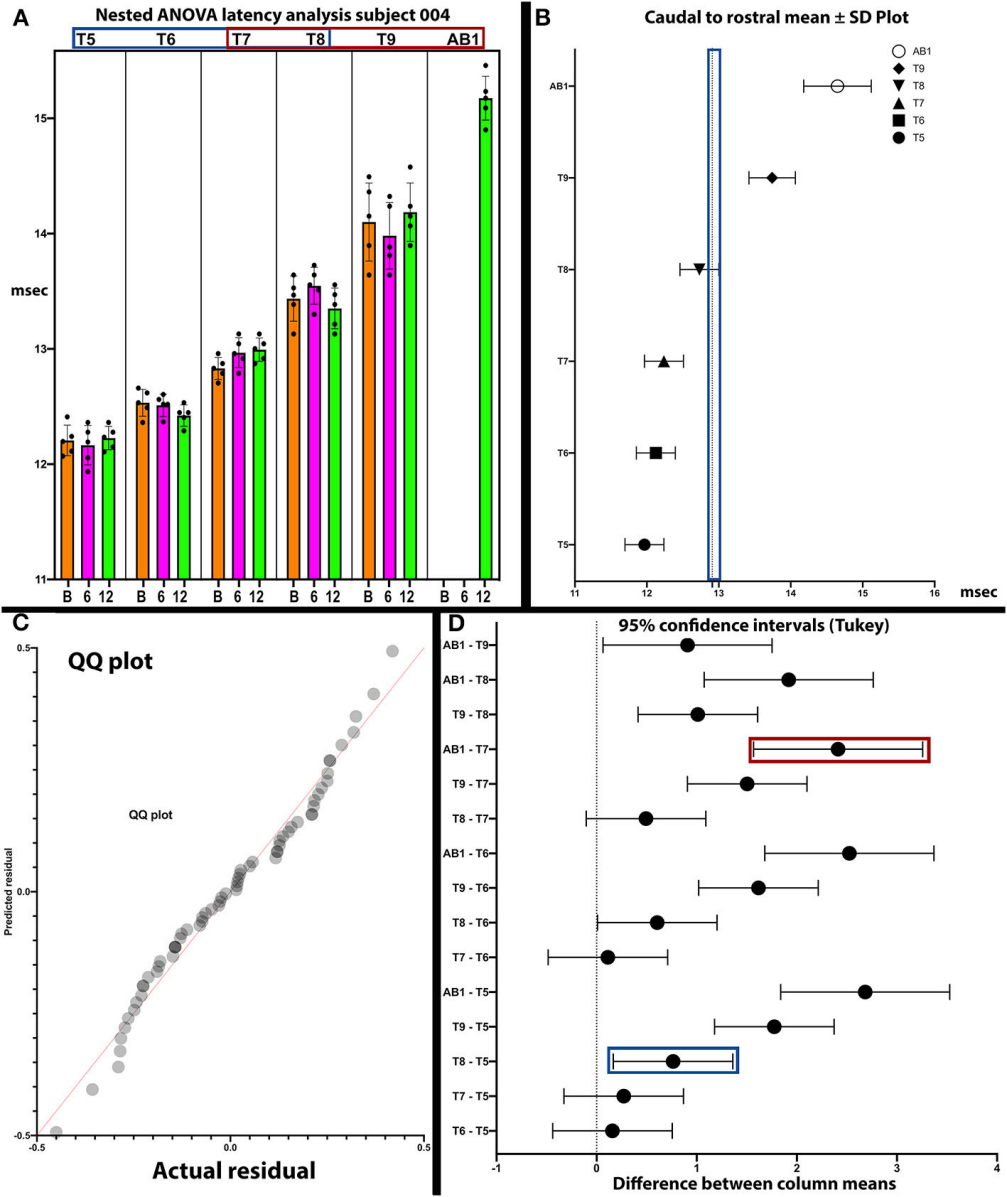
Analysis of latencies trends in subject 004. The latencies are shown in **(A)**. In **(B)**, the standard deviations are indicated and those of T9 and AB1 are larger, possibly due to varying conduction. In **(C)**, the QQ plot is compatible with data sphericity. In **(D)**, the 95% confidence intervals for thoracoabdominal mean separations is much larger for T7-AB1 (red box) than for T5–T8 (blue box). These differences in latency data indicate that a greater per level delay in transmission to the 4 levels of T7/8/9/AB1 than to T5/6/7/8. Although MEP conduction is present there is a differential and a notable difference in the amplitude trend (Figure 8) between these levels.

Summary thoracoabdominal MEP amplitudes and latencies are shown in Figure 8. Unexpectedly, IC MEPs were detectable below the NLI in all participants studied, even at the pre-transplant time point. The MEP latencies increase with successive caudal levels, and the SD size was larger in the most caudal levels: IC 8, IC 9, and AB1 (Figure 9). This may indicate more variable MEP transmission to these intercostal nerves. The recorded latencies for upper thoracic ICs are close to those reported in uninjured persons using direct scalp stimulation (11.1 to 14.5 ms) and longer than those reported from nearby muscles such as pectoralis major, latissimus dorsi (<10 ms) (55) but similar to serratus anterior (85). It is also important to consider the possibility of volume conduction from the diaphragm; points mitigating this possibility are the low baseline amplitudes, spasticity in the signals, evidence of fatigue, and early lack of signal continuity.

For the rectus abdominus, the recorded MEPs were small with modest changes over time. The participant's small MEP amplitudes at IC 8, IC 9, and AB1 were distinctly different from the controls (Figure 2). At baseline, 006 and 008 had smaller amplitudes as compared to 004 and 009, possibly related to resolving spinal shock. The amplitudes were modestly increased by the 6 and 12-month timepoints except in participant 004, where they declined. Overall, the MEP amplitudes were larger in IC 5, IC 6, and IC 7 than at lower levels. The most notable intercostal transition was for 001-T3 at endpoint with a right IC 6 MEP of 1.9 ± 0.2 mV at 12.1 ± 0.12 ms, no detected waveform from IC 7, and an IC 8 MEP of 0.13 ± 0.09 mV (14.6X less) at 13.8 ± 0.3 ms, with detectable but small IC signals to T10.

### Assessment for an Electrophysiological Thoracic Motor Level

#### Intercostal Latency and Amplitude

In *posthoc* analyses, we explored the possibility that MEP and EMG recordings might provide evidence of a transitional motor level from greater to lesser amplitudes and an increased delay in latency in the thoracoabdominal study participants. In Figure 9, a plot of subject 004 whose sensory NLI was T1, the IC 9, and AB1 SDs are larger, and the 95% confidence interval is larger for the IC 7-AB1 levels as compared to the IC 5-IC 8 levels. The column means ± SD also cluster with a latency breakpoint at ≥13 ms. This type of analysis may provide a method to examine for a transitional motor level between greater and lesser innervated IC muscles.

**Figure 10:**
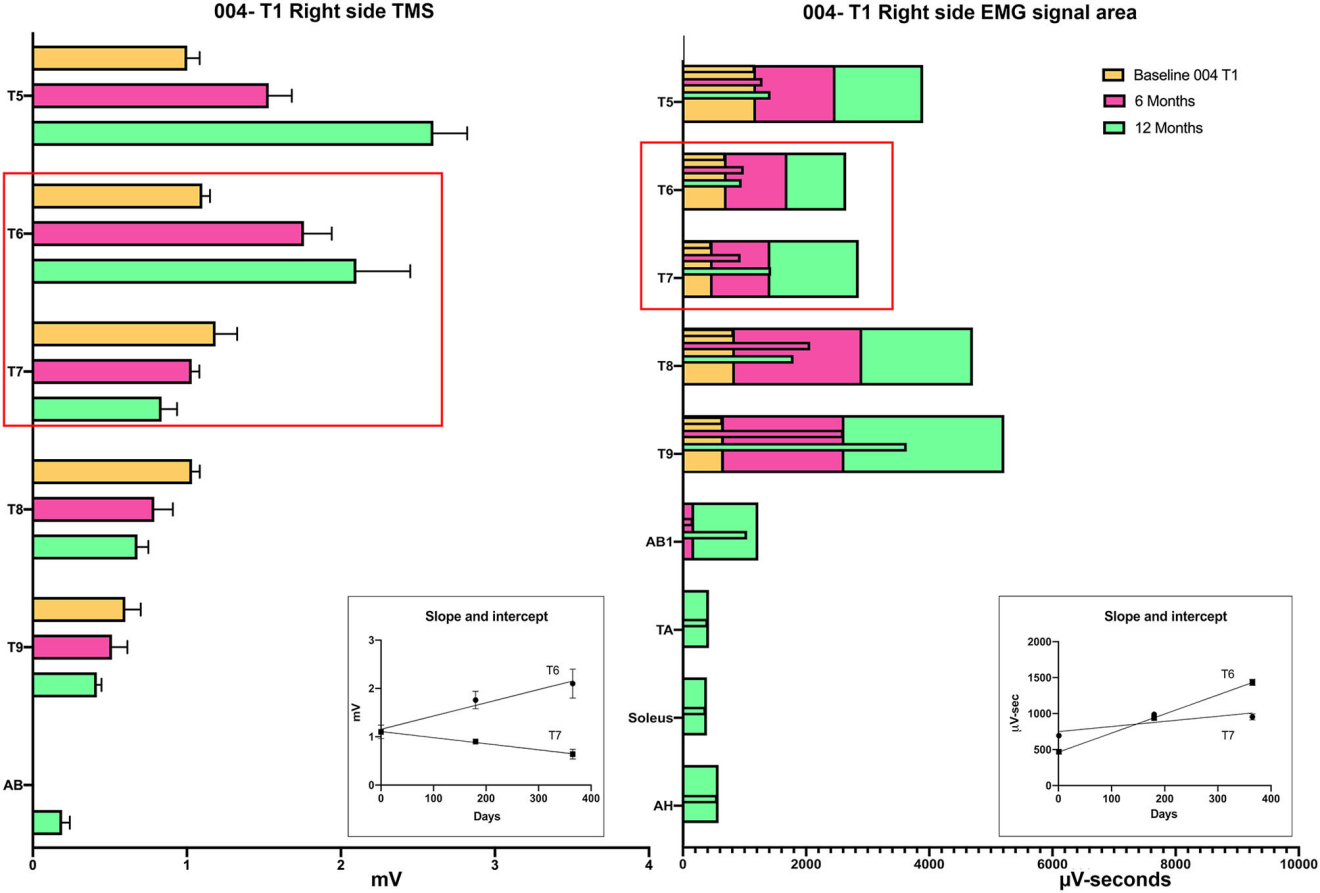
Comparison of the recovery trend for MEPs and held-breath EMGs. In the left panel, the change in MEP amplitude from baseline to 6 and 12 months is illustrated as stacked bars per level. The red box around T6 and T7 emphasizes that the trend for amplitude recovery seen in T5 and T6 is different in T7 and below where the amplitudes are decreasing with time. Linear regression is used to analyze the temporal change in T6 that has a positive slope wheras that of T7 has a negative slope. In the right-side panel, the area of the breath hold in μV-seconds is displayed and shows an opposite trend whereby all levels but especially T8 and T9 show increases over the assessment time points (seen as positive slopes in linear regression analysis). This supports a conclusion that the breath-hold areas indicate improved chest bellows function and not necessarily better supraspinal innervation.

Intercostal EMG and MEP amplitudes. The IC MEP amplitudes in the most caudal levels, IC 8 and IC 9, were much smaller than the more rostral levels and differed from the control participants. In comparison, we observed that the held inspiration IC EMG amplitudes in the participants generally improved across the assessments. At 12 months, they were larger and more stable regardless of the NLI. In the controls, there was a positive correlation between the MEP and the held EMG amplitudes (*R*^2^ = 0.668, Pearson correlation *p* < 0.001). However, no such correlation was found in the participants. The held-breath EMG improved at all levels, but the MEP amplitudes did not improve in proportion. Figure 10 shows the serial MEP compared to EMG changes across the B, 6M, and 12M time points. In this participant, 004, IC 5, and IC 6 IC show increasing MEP amplitudes whereas IC 7, IC 8, and IC 9 did not improve and had lower amplitudes and 6 and 12 months. In a linear regression model, the IC 6 MEP had a positive slope for amplitude recovery over time, but IC 7 had a negative slope. For the same two levels, the held breath EMG changes showed a positive slope for amplitude recovery.

### Intercostal Spasticity

We had not anticipated the observation of multiple spasms in the IC recordings, even when there was a MEP present. This spasticity may also contribute to the identification of a thoracic motor level. Participant 006-T4 had the highest incidence of apparent spasms detected in the thoracoabdominal exams, most notably at the endpoint. Occasionally, an entire contraction had a spastic appearance at one level while the other simultaneously recorded levels did not (Supplementary Figure 8). Some unusual activations were separated by pauses resembling cutaneous silent periods (86). The amplitude of IC spasms was generally in the 1 mV range. Although the spasms were most prominent at the lower levels, they were also occasionally detectable at the most rostral recorded level, IC 5. In a prior SCI study, Guttman and Silver reported that large inspirations could elicit intercostal spasms (87). One conclusion from these observations is that voluntary activity and spasticity can coexist in the same IC signal.

### Brachial Plexus Injury Follow-Up

Thoracic SCI often presents with concomitant polytrauma due to high-velocity injury mechanisms (88). Participant 002-T6, injured in a motorcycle crash, sustained a right brachial plexus, rotator cuff, and clavicle injury along with scapular and rib fractures. For this participant, we recorded sequential arm muscle improvements using MEPs and correlated these to the evolving motor scores. At the baseline time point, the right arm ECR, BB, and TRI had markedly decreased amplitudes consistent with upper/middle trunk injury; latencies were in the normal range. These three muscles showed amplitude improvement into the mV range at the 12-month assessment and a trend toward shortened latencies. Detailed findings are reported in Supplementary Figure 9. Despite almost normal motor scores at the 12-month endpoint, the affected muscle MEP amplitudes remained below normal. This provides an interesting internal control and perspective of peripheral vs. central nervous system injury (28) and the timing and extent of recovery.

### SSEPs and Sensory Levels

**Figure 11:**
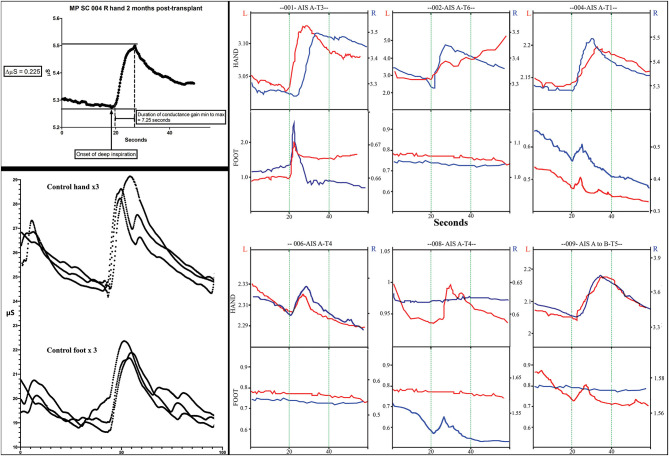
Skin conductance response traces. The measurements of electrodermal activity in microSiemens (Y-axis) and seconds (X-axis) are illustrated in the left upper panel for subject 004. Replicates of three are shown in the lower panel for a control subject for both the hand and foot. Smaller notches are caused by additional breaths. Note that the traces are larger in the control subject. In the right panel, representative traces from all subjects for both right and left hands and feet are shown. The GO command to take a deep breath(s) is given at second 20 during visualization by the subject. Up to three deep sequential breathes were taken as a stimulus. A deep breath typically caused a rapid increase increase in conductance followed by a slower reduction in conductance.

The stimulation protocol and SSEPs recorded during the trial are summarized in Figure 11 for a representative SCI participant and a non-injured individual and quantified and segregated into SSEP components in Table 4. No conclusively positive cortical SSEPs from tibial nerve stimulation were observed at any assessment time-point (89). Positive control recordings at sites below the injury level (tibial nerve in the popliteal fossa and the spinous process of T12) and technique controls (median nerve SSEPs) are shown. Uniformly positive control signals indicate the nerves were viable, and axonal conduction was triggered. Conduction latency and amplitude from the tibial nerve to N22 (T12) was comparable between the subjects and controls. We noticed external stimuli such as noise, feeling cold, and anxiety could negatively influence the quality of the brain's surface potentials. Thus, SSEP recordings occurred in quiet darkened rooms on comfortable beds with blankets, relaxation was induced, and participants usually fell asleep while being stimulated. During the early stimuli, we sometimes saw convincing waveforms emerging at delayed latencies, but after additional averaging, these became unclear or absent. Regarding sensory levels, at endpoint, a sensory ZPP below the NLI was found in caudal dermatomes [001 (+1), +1 002 (+1), 004 (+2), 006 (+3), 008 (+2, left only)]. Participant 009, who had converted to AIS B, had residual sensation [light touch only] (90) to the right T9 and Left T10 at the six and 12-month assessments (Supplementary Figure 2), although these dermatomal findings differed between examiners as is not uncommon (91, 92).

**Table 4:** Summary of observed SSEP values.

**Median nerve**				**N9/EP**		**P14**		**N20**	
				**Mean**	**SD**	**Mean**	**SD**	**Mean**	**SD**
	Left	Latency (ms)	Controls	9.90	0.31	13.25	0.66	18.35	0.61
			Subjects	↑10.40	0.57	↑14.85	0.15	↑20.40	0.79
		Amplitude (mV)	Controls	9.18	2.74	0.85	0.29	2.13	1.07
			Subjects	↓5.08	1.71	2.50	0	2.33	0.38
	Right	Latency (ms)	Controls	9.93	0.22	13.16	0.52	19.22	1.32
			Subjects	↑10.55	0.61	↑14.80	0.40	↑20.88	0.83
		Amplitude (mV)	Controls	5.83	4.85	1.18	0.68	1.66	1.09
			Subjects	↓4.73	1.56	1.35	0.35	2.58	0.54
**Tibial nerve**				**N22/T12**		**P37**		**N22–P37**	
				**Mean**	**SD**	**Mean**	**SD**	**Mean**	**SD**
	Left	Latency (ms)	Controls	21.38	1.68	39.23	1.18	17.85	2.75
			Subjects	↑23.50	0	0		0	
		Amplitude (mV)	Controls	0.85	0.52	0.90	0.27		
			Subjects	↓0.50	0	0		0	
	Right	Latency (ms)	Controls	22.02	0.85	38.52	1.58	16.66	1.06
			Subjects	↑24.20	0	0		0	
		Amplitude (mV)	Controls	1.12	0.27	0.94	0.23		
			Subjects	1.20	0	0		0	

*Control values include single session recordings from 5 non-SCI individuals, 10 SSEPs are averaged per nerve. Subjects' values include average of all 6 subjects through 4 different sessions (pre-transplant, 2, 6, and 12 MPT), 48 SSEPs are averaged per nerve. P37 was negative throughout the trial in all subjects*.

### Galvanic Skin Response

**Figure 12:**
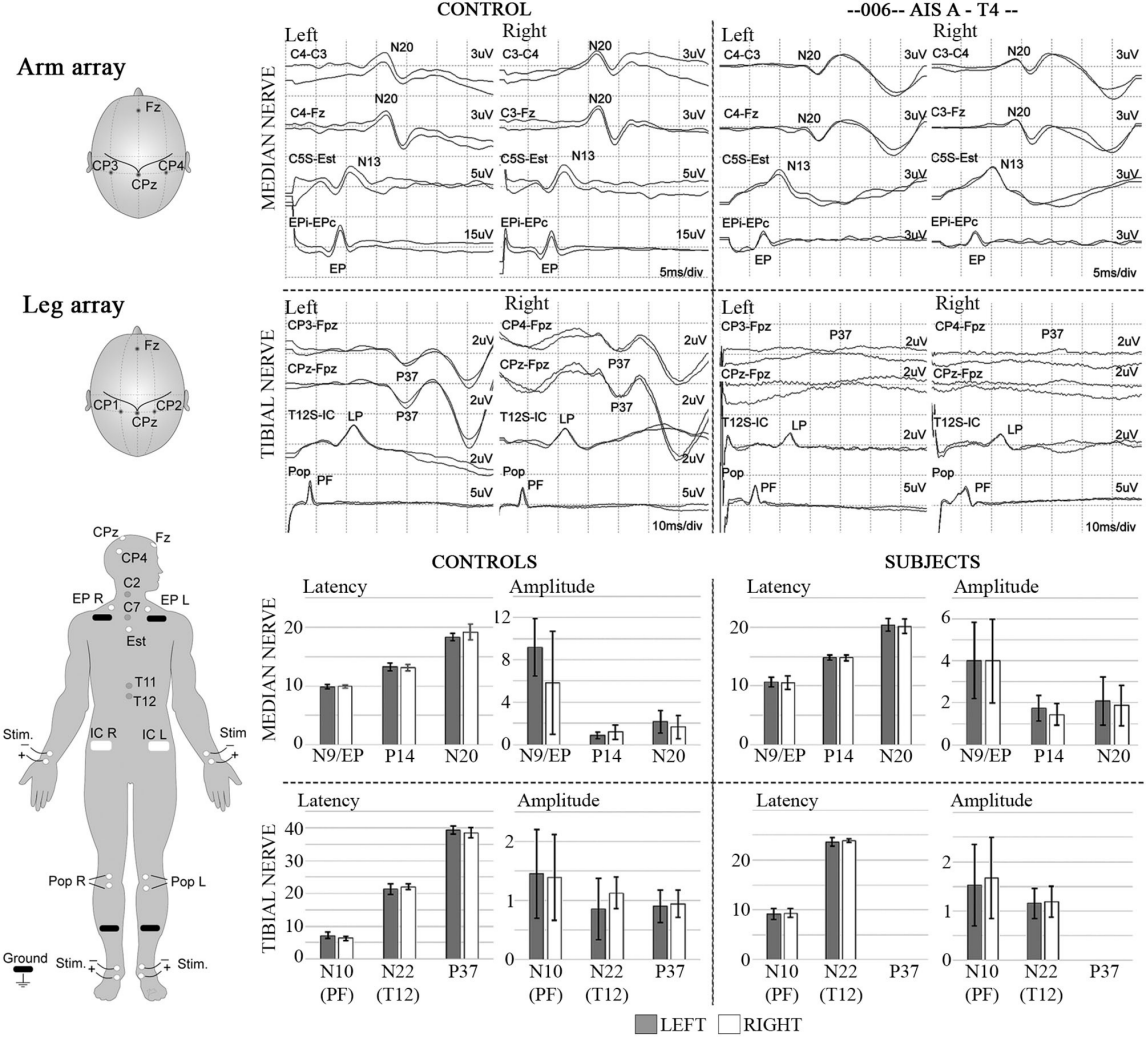
SSEPs. Left diagrams show recording and stimulation sites for SSEPs. Monophasic square-wave electrical pulses, duration 0.2ms, Intensity 15-30mA (2X motor threshold), rate 3Hz (autocorrected) were used. Representative SSEP recordings from a control and a T4 SCI subject are shown. Standard recording sites for median nerve include compound action potential for ipsilateral Erb's point (EP), cervical potentials recorded at the C5 spinous process (N13), and the cortical somatosensory component (N20) using C3/C4-Fz and C3-C4/C4-C3 derivations, respectively. For tibial nerve stimulation the compound action potential at the popliteal fossa (PF), a lumbar potential recorded over the T12 process (LP), and the cortical somatosensory component (P37) using CPz-Fpz and C3/C4-Fpz derivations, respectively. Scales are indicated individually. These results were consistent within all subjects in the trial, where the last point of positive recording from tibial nerve stimulation was obtained from the spinous process of T12. Lower panels show mean and standard deviation values for SSEPs latencies (ms) and amplitudes (μV) in controls (n = 5) vs. the six subjects recruited in the trial, the P37 component is absent in all subjects.

We used the GSR to test for a change in sweating and, thus, skin conductance in the hands and feet as an indicator of changing autonomic activity. Although the sympathetic skin response (SSR) is often evoked via peripheral nerve stimulation, we chose deep inspirations (93) as these stimuli gave large amplitude changes in healthy volunteers, were used in another trial we reported (12) and have been used in other SCI studies (94, 95). The summary findings are shown in Table 5. The baseline conductance was always greater in the upper vs. lower extremities. The smallest positive reproducible signal was in the left foot of participant 009-T4 at 2M post-transplantation of 0.03 μS over a 4.1 second period. At baseline, we were only able to detect reproducible upper-extremity GSRs from 3/6 participants 004-T1, and 008-T4, right side only, and 009-T4, bilaterally, and some conductance changes were minimal in magnitude. By 12 months, all participants had at least one positive hand (Figure 12). For the lower extremities, none of the participants had detectable conductance changes at baseline, and two (001-T3, 004-T1) had bilateral and two unilateral (008-T4, 009-T5) lower limb deep breath induced conductance changes at the final endpoint. Some signals (001-T3) had an unusually steep and abrupt slope and did not show the falling baseline observed in the upper extremities during relaxation breathing. No participant had GSR amplitudes into the normal range (Figure 12).

**Table 5:** Galvanic skin response summary data.

**Subject**	**001–T3**		**002–T6**		**004–T1**		**006–T4**		**008–T4**		**009–T3**	
	**U**	**L**	**U**	**L**	**U**	**L**	**U**	**L**	**U**	**L**	**U**	**L**
Baseline	N	N	N	N	P	N	N	N	P	N	P	N
2M	N	N	N	N	P	N	P	N	P	N	P	N
6M	P	N	P	N	P	N	P	N	UA	UA	P	N
12M	P	P*	P	N	P	P*	P	N	P	N	P	N
		**Left hand**			**Right hand**		**Left foot**			**Right foot**		
		**ΔS**	**ΔSec**		**ΔS**	**ΔSec**	**ΔS**		**ΔSec**	**ΔS**		**ΔSec**
001	Base	0	0		0	0	0		0	0		0
	2M	0	0		0	0	0		0	0		0
	6M	0.06	7.8		0.08	9.7	0		0	0		0
	12M	0.107	10		0.22	10.19	1.02		1.93	0.012		1.31
002	Base	0	0		0	0	0		0	0		0
	2M	0	0		0	0	0		0	0		0
	6M	0.04	4.1		0.06	7.9	0		0	0		0
	12M	0.72	6.5		2.56	5.4	0		0	0		0
004	Base	0	0		0.08	3.4	0		0	0		0
	2M	0	4.1		0.23	7.25	0		0	0		0
	6M	0.175	0		0.19	6.7	0		0	0		0
	12M	0.068	14.3		0.26	10.63	0.007		6	0.04		5
006	Base	0	0		0	0	0		0	0		0
	2M	0	0		0	0	0		0	0		0
	6M	0.21	18		0.13	12	0		0	0		0
	12M	0.03	6.4		0.22	14	0		0	0		0
008	Base	0	0		0.3	13.32	0		0	0		0
	2M	0.36	12.1		0.27	11.8	0		0	0		0
	6M	UA	UA		UA	UA	0		0	0		0
	12M	0.07	7.15		0.34	5.4	0		0	0.09		6.2
009	Base	0.04	8.2		0.06	6.8	0		0	0		0
	2M	0.07	6.9		0.08	7.3	0.03		4.1	0		0
	6M	0.09	7.1		0	0	0		0	0		0
	12M	0.12	13.43		0.3	13.6	0.07		6.3	0		0

U, Upper extremity; L, Lower extremity; P, Positive; N, Negative; UA, unable to assess; M, Months. Negative recordings are in red. Numerical values of recordings marked as positive are given in lower portion of the table for baseline and 12–month assessments as ΔS, Total conductance change in microSiemens: Total duration of observed slope change/ signal in seconds. P^*^-The signals recorded from the feet of 001 had a very abrupt slope that may be seen in Figure XX.

### The Participant Experience During Electrophysiology

The complete EP testing protocol required up to 6 h. All participants were accompanied by their spouses or family members. During these prolonged interactions, time gaps were used to provide participant and spouse education regarding the test's purposes and to discuss other issues such as pain, spasticity, sleep, and recovery from other associated injuries. We successfully taught the participants to minimize the baseline signal activity through calm, controlled breathing exercises before their voluntary effort acquisition windows, somewhat like biofeedback. Since the participants often fell asleep during SSEP testing, we considered these techniques useful. Despite the discomfort associated with TMS, we found the participants to be interested in their testing, especially when we requested visualization and voluntary reinforcement maneuvers to amplify MEPs or EMG.

## Discussion

In this subacute trial of aSC transplantation, we serially tested for electrophysiological changes of the motor, sensory, and sympathetic systems above and below the sensory-defined neurological level of injury over a 12-month period. The most sensitive assessment was voluntary EMG, detected even when MEPs remained negative. The lack of a control non-transplanted SCI group prevents us from concluding that Schwann cell transplantation contributed to the observed circuitry changes. Further, there has been limited systematic longitudinal study of electrophysiological changes in the thoracic AIS A paraplegic population for comparison. This is likely because of the poor prognosis for recovery from these injuries. Recent discoveries have shown that participants with a clinically complete neurological exam can exhibit volitional control of movement during epidural and transcutaneous stimulation (96–98) raising the importance of limited residual axonal connectivity.

### Study Limitations

The enrolled participants came from geographically diverse locations and returned to home after acute care, transplantation, and rehabilitation. Follow-up visits thus required travel and expense and were limited to 3 days. While it is possible the neurophysiologic data might have been further enriched by repetitive testing sessions, that was not feasible in this clinical trial. It is unlikely future SCI clinical trials of drugs and biologics with industry sponsors will be open to incorporate neurophysiologic testing unless they can conduct it with reasonable efficiency.

We focused on trunk, abdomen, and leg signals taking care to use identical recording sites between sessions (73). The absence of initial responses in the legs to 100% TcMEP stimuli precluded the determination of a “hotspot,” the optimal coil angle from perpendicular (99), a minimal stimulation threshold, and an MEP/CMAP ratio. While intercostal needle recording may have been more specific, surface intercostal recordings have been validated against needle recordings (87). Challenges related to obtaining and interpreting IC signals are discussed below. With more channels, it would have been valuable to record from parasternal ICs and posterior paraspinal muscles (36, 68), which would aid in further characterizing truncal connectivity. Specific trunk function testing was not performed, nor was spirometry.

### The Natural History of Clinical Neurological Recovery

The AIS conversion rate of 1/6 participants is 16 percent, similar to the natural history we recently reported from multiple datasets (9). The 40-day delayed assessment *baseline* before transplantation would be associated with a further reduction in expected AIS conversions (100). Our detection of improved electrophysiologic connectivity in this study cohort adds to evidence of safety and offers insights into assessments to be refined for future SCI trials, in particular, for the thoracoabdominal region.

### Limitations of the ISNCSCI in Paraplegia

The ISNCSCI examination for SCI classification is part of the inclusion/exclusion criteria of virtually all therapeutics trials in SCI. The ISNCSCI provides inexpensive reproducible testing and predicts long-term functional outcomes (101, 102). Recognized ISNCSCI limitations include (1) The thorax and abdomen have only a frontal sensory assessment (103), the back is not evaluated, (2) Sensory testing of a dermatome is conducted at specific anatomical points; thus, patches of sensations over other parts of the dermatome are not captured (22), (3) Motor function is only tested for the arms and legs, and (4) There is inter and intra-examiner variability, and continued training is necessary to reduce this bias (104, 105). Here, all the participants studied had detectable EPs below the ISNCSCI defined neurological level of injury, and 5/6 could evoke voluntary EMG from leg muscles at 12 months post-transplant despite AIS A status. These findings add to the evidence that some voluntary motor activity may be observed in AIS A classified individuals under specific conditions (97, 106, 107).

### What Is the “Level” of a Thoracic Injury?

It has been accepted that the thoracic sensory level is a *surrogate* for the thoracic motor level based on a *dermatomal conception* of trunk function, defining the NLI. Other than sensory function, there is support for the “level” concept from studies of autonomic deficits (108, 109), blood pressure instability (110), and immune dysregulation (111, 112). However, such studies use broad ranges such as above or below T6, upper vs. lower thoracic, and thoracic vs. cervical (113) and not discrete segmental levels. Others have reported evidence of motor activity below the NLI for paraspinal (114) and IC muscles (68, 72). Cariga et al. reported a dissociation between the sensory NLI and posterior paraspinal MEPs (114). IC and abdominal MEPs have even been recorded from high-level incomplete quadriplegics (71).

At the pre-transplant baseline, all participants had an NLI of T6 or higher, whereas both MEPs and thoracoabdominal EMG were detected below this level. The breath-hold EMG amplitudes consistently improved between the initial and final testing sessions. The IC MEPs mainly had sequentially decreasing amplitudes and increasing latencies from rostral to caudal levels (Figure 8), while the uninjured control participants had no rostral-caudal amplitude trend (Figure 2). While some of the observed IC signals might be contaminated from the serratus anterior, the sequential differences in latency, small signals, and electrode positioning anterior to its attachments should have minimized this artifact as reported by others (87). Further, we would not expect the obvious EMG fatigue observed since the serratus is innervated by the long thoracic nerve from the brachial plexus, nor would we see EMG evidence of spasms. The rectus abdominis recordings obtained using needle electrodes are not likely to be contaminated from other nearby muscles. Thus, we think these signals indicate real innervation.

In the absence of SCI, the external ICs are active during effortful inspiration; they raise the chest cage and synergistically stabilize it against diaphragm motion. Increased EMG activity (115, 116) is recorded in sequence from rostral to caudal (117). Activation is earlier if the tidal volume is larger (>1.0–1.5 L), especially for the lower ICs such as IC 7 and IC 8. For this reason, we asked participants to initiate a deep breath and then a static breath-hold. We anticipated that during the static hold, the tendency of the chest to recoil and exhale would require maintenance of external IC activity. Whereas, we used surface electrodes, Whitehall and Feroah used wire electrodes placed close to the muscles via needles (117). This is more specific for IC discharge but carried some risk, and they did report a post-procedure chest hematoma. In a study of chronic paraplegics, Frostell and colleagues examined for IC motor unit potentials (MUPs) above, at, and below a neurological level by monitoring IC activity during two conditions, neck flexion and lower extremity spasms (70). In some participants, they observed spontaneous discharges compatible with denervation hypersensitivity at mid-thoracic injury levels. At rostral ICs, they observed evoked MUPs during neck flexion. Below, they did not see activity during neck flexion but found that leg spasms could cause an IC MUP discharge. They did not assess MEPs or the association of MUPs with breathing. We also observed occasional IC spasms as reported by others from EMG observations (87, 118). In this study, we did not observe a lower extremity muscle spasm to generalize into the intercostals, but they were triggered by breathing. By correlating the integrity of IC MEPs and EMG observation of spasticity, we propose that IC spasticity and voluntary activity may occur at the same time.

For breathing function, the NLI concept is too simplistic; first, the sensory and motor systems exhibit fundamentally different spinal cord organization. Second, supraspinally innervated large muscles attach to the chest wall and contribute to its motor function. Third, chest and abdominal wall mechanics are linked and cannot be isolated to a single IC muscle (119). The innervation of respiratory muscles is complex and multi-segmental; propriospinal interneurons in IC motor neuron pools involved in breathing may span several levels and contribute to post-injury plasticity (120). In studies of cats, IC motor neurons have been shown to receive input from propriospinal interneurons with processes descending 2–4 levels (120, 121). In people, Butler et al. provided evidence for rostral-caudal neuromechanical matching in which ensemble networks of premotorneuronal spinal neurons link IC activation during voluntary inspiration (122). Further, there are IC afferent connections that regulate activity in other respiratory muscles (123). Intercostal nerve anatomy may also be a factor to explain our observation of IC and rectus MEPs below the sensory level-defined NLI. Branches from ICs innervate below their segmental origin (124), and the rectus abdominis receives innervation from IC 6 or IC7 (125, 126).

### TMS-Triggered Activations Below the Neurological Level of Injury

Evidence for small amplitude or delayed latency MEPs in motor-complete chronically injured individuals has been reported (106, 127–129). In prior studies, a prognostic significance has been demonstrated for the detection of *early* positive MEPs after SCI for hand function and ambulation (130). In another study, the predictive value of the MEP amplitude was stratified into those above and below 0.1 mV (23). Here, only late time-point limb MEPs were observed (≥ 6-months post-transplant), and recovery of joint movement was not observed. We found substantially prolonged TA and AH latencies possibly attributable to residual demyelination (131–133) in spared fibers that may interfere with spatial and temporal summation. Circuit changes in terms of corticospinal terminations on intrinsic spinal interneurons may also be present. Dimitrijevic et al. described different descending motor latencies in non-injured persons, identifying a prolonged MEP (latency of 72 ms) in the TA, which they postulated was due to descending fibers transmitting through spinal cord interneuronal circuits (134). Due to the time constraints of a therapeutics clinical trial, we did not perform threshold MEP testing, which may enhance the ability to identify a spinal level at which the required stimulation threshold changes, as reported by Ellaway et al. (135) to indicate an injury level transition. We observed that whereas rostral MEPs increased in amplitude with time, lower level MEPs showed less change. This difference may represent reduced descending connections, longitudinal change due to disuse, and reorganization of afferent inputs to favor those from below the injury.

### EMG Detected Leg Muscle Activity Below the Level of Injury

The delay in voluntary EMG onset of 1.4 to 3.3 s to TA and AH is reminiscent of studies of epidural stimulation where the intention to move the toe is delayed during initial training (107). This delay may reflect pathway changes such as new circuits or incomplete myelination and attempts to recruit supraspinal inputs that have not been used after SCI. McKay et al. (76) reported that EMG activation can be initiated at command but may not be sustained during the full effort window, as we observed. While MEPs provide unequivocal stimulus-dependent evidence of a connection to muscle, our data suggest voluntary EMG is a more sensitive test to detect residual circuit connectivity in the legs. Tables 3, 6 shows summary data for all participants, including ISNCSCI, EMG, MEPs, and SSEPs. The latency of MEPs to leg muscles was considerably higher than average but showed reproducible waveforms (Figure 4) and are in agreement with prior SCI studies (136, 137) but longer and with less amplitude than detected in motor incomplete SCI (138). Baclofen did not preclude MEP detection in agreement with the report by Nardone et al. (139). All participants were on Baclofen for most of the study (Table 2).

**Table 6:** Summary data of clinical (ISNCSCI) and electrophysiological results.

**Subject**			**001**		**002**		**004**		**006**		**008**		**009**	
**Side**			**R**	**L**	**R**	**L**	**R**	**L**	**R**	**L**	**R**	**L**	**R**	**L**
ISNCSCI	AIS	B	A		A		A		A		A		A	
		6	A		A		A		A		A		B	
		12	A		A		A		A		A		B	
	Motor level	B	T3	T3	T6	T6	T1	T1	T5	T4	T4	T4	T4	T4
		6	T1	T1	T6	T6	T2	T2	T5	T4	T3	T3	T4	T4
		12	T1	T3	T6	T6	T2	T2	T4	T4	T3	T4	T4	T4
	Sensor y level	B	T3	T3	T6	T6	T1	T1	T5	T4	T4	T4	T4	T4
		6	T1	T1	T6	T6	T2	T2	T5	T4	T3	T3	T4	T4
		12	T1	T3	T6	T6	T2	T2	T4	T4	T3	T4	T4	T4
	MZPP	B	T3	T3	T6	T6	T1	T1	T5	T4	T4	T4	T4	T4
		6	T1	T1	T6	T6	T2	T2	T5	T4	T3	T3	*T4	*T4
		12	T1	T3	T6	T6	T2	T2	T4	T4	T3	T4	*T4	*T4
	SZPP	B	T3	T3	T7	T7	T4	T4	T6	T5	T5	T5	T4	T4
		6	T4	T4	T7	T7	T4	T4	T7	T5	T4	T5	T10*	T11*
		12	T4	T4	T7	T7	T4	T4	T7	T7	T3	T6	*T9	*T10
	NLI	B	T3		T6		T1		T4		T4		T4	
		6	T1		T6		T2		T4		T3		T4	
		12	T3		T6		T2		T4		T3		T4	
	VAC/ DAP	B	No		No		No		No		No		No	
		6	No		No		No		No		No		DAP+	
		12	No		No		No		No		No		DAP+	
B	SSEPs		Negative		Negative		Negative		Negative		Negative		Negative	
2	SSEPs		Negative		Negative		Negative		Negative		Negative		Negative	
6	SSEPs		Negative		Negative		Negative		Negative		Negative		Negative	
12	SSEPs		Negative		Negative		Negative		Negative		Negative		Negative	

*Baseline, 2, 6, and 12 months. AIS, ASIA impairment scale grade; VAC, voluntary anal contraction; DAP, Deep anal pressure; MZPP, Motor zone of partial preservation; SZPP, Sensory zone of partial preservation; ZPPs are defined as most caudal levels with any clinically detectable innervation meeting ISNCSCI criteria. NLI, Neurological level of injury; SSEP, somatosensory evoked potential; MEP, motor evoked potential; EMG, Electromyography. R, Right; L, Left; T#, Thoracic segment*;

* *At 6 and 12 months subject 009, had scores of 1 for light-touch at the S4–5 right and left dermatomes and positive DAP, which converts the subject to AIS grade B incomplete, thus levels given for ZPPs at final end point are assumed in the absence of sacral dermatomes or DAP. 009 also had some sensation as low as T10*.

### The Effect of Reinforcement Maneuvers on EMG Amplitude

During this study, attempts to record EMG and MEP activity were performed with and without “reinforcement” maneuvers. EMG amplitudes were markedly potentiated by the three reinforcement procedures used: the classic finger interlocking Jendrassik maneuver (JM) (140), jaw-clenching (65), and deep breathing. Had we not used reinforcement maneuvers, our detection of voluntary EMG activation in the legs would have been more difficult. The most prominent effect was observed in participant 009-T4. The within-session difference between unreinforced and reinforced MEPs for AH amplitude was 8.8 × (54 to 475 μV) for C1 stimulation and 6.7 × (71 to 479 μV) for Cz stimulation (Figures 5, 6). These magnitudes and the associated reduction in latency are similar to those previously reported by (65) and appeared to be effort-dependent (141). The mechanism by which these facilitating maneuvers amplify muscle output remains a topic of investigation we did not specifically explore. Reinforcement resulted in a larger amplitude increment in the study participants than in the controls. Reinforcement was initiated just before either the TMS pulse or attempted voluntary contraction (142). The reinforcement maneuvers sometimes triggered leg spasms in participants 004 and 009, but these had much higher amplitudes and visual evidence of muscle and limb contraction. Spasticity and residual spared spinal cord have recently been linked; spasticity may be a clue to participants with more retained trans-lesional connectivity, as appeared to be the case in these two participants (143).

### SSEPs

We found no reliable evidence of sensory transmission from the tibial nerve to the cortex despite evidence of motor connectivity, which causes us to question technical aspects of SSEP acquisition. After SCI, techniques of time-locked ensemble *averaging* may not be optimal if the signals are small or they vary in latency as may axons undergoing spontaneous repair (144). A second-order blind source technique (145) or a time-frequency analysis might be better suited to characterize signals exhibiting variable time and amplitude (146, 147). This was demonstrated for participants with cervical spondylotic myelopathy recovering after decompression surgery (145). These methods require that single-trial SEPs are visible. SSEPS are more likely to be observed in trials of cervical injury where *both* the ulnar and median nerve can be stimulated in motor-complete participants (34). Given the substantial delay observed with MEPs, sensory conduction delay is also likely, and the window settings should be extended. Another method to track segmental sensory changes is dermatomal SSEPs (148). Pre-motoneuronal IC motor innervation may be distributed across levels. Dorsal root ganglia, however, receive segmental sensory fibers within the intercostal nerve. Direct comparison of dermatomal SSEPs to IC MEPs may further inform our understanding of the organization of IC nerves.

### Thoracic Injury and Autonomic Impairment in the Upper Extremities

To gain insight into autonomic connections and responsivity in the participants, we tested the GSR, expecting the upper extremities to serve as a control for the legs. The GSR is a slow conductance change at the skin surface due to eccrine gland activity after an unexpected stimulus (149). We expected positive signals in the hands of those participants with injuries below T3 (150). However, three participants (001-T3, 002-T6, and 006-T4) lacked detectable GSR changes at baseline assessment. This may result from several factors: (1) Anatomy, the cervicothoracic stellate ganglion receives innervation from multiple thoracic levels at least as low as T4 (151). (2) Physiology, the period of spinal shock following complete SCI, may disrupt the reflex for several weeks (152, 153). (3) Pharmacology, conductance may be decreased due to secondary antagonism of M1-M3 sweat gland receptors caused by anticholinergic medication (oxybutynin) used to reduce neurogenic bladder symptoms. We did not observe a clear relationship between injury level and the presence of an upper extremity GSR. By the 12-month post-transplant assessment, 4 participants had a reproducible Δ GSR in the feet, although small in amplitude. GSR appears to be a sensitive technique.

### The Value of Electrophysiology Studies in Complete SCI

Electrophysiology studies have more frequently been conducted in chronic SCI participants (128, 154) or intraoperatively (155). Evidence of transmission through the injury region establishes that apparent clinical completeness does not preclude residual circuit connections (156). Recent neuromodulation studies have provided evidence that participants whose clinical exams are designated as “complete” may respond to ES by displaying voluntary motor activity (157, 158). In the past, this connectivity has been considered sub-threshold or “*dyscomplete*” (159, 160), but the ES studies are redefining the “threshold” for voluntary activation and its significance in combination with rehabilitation training (97). In this Phase 1 study, electrophysiology was the only outcome that showed quantifiable neural connectivity changes within each participant. Convincing motor signal (EMG, MEP) recovery in the legs was not detected until 6 months after transplant, although 008-T4 and 009-T4 had tiny EMG signals at 2 months post-transplant (Supplementary Figure 5). This time course is similar to that reported in a trial of neural stem cell transplantation carried out as a single-center study (36) and using the functional neurophysiology assessment—(FNPA) (161) in a longitudinal cohort. Regarding other possible correlates, tissue bridges, visible on MRI, have been reported to correlate with EPs (31) In this study, tissue bridges on MRI were <1.5 mm (32).

#### Future Directions

For future studies, we recommend research to validate our findings in people with paraplegia to discriminate thoracoabdominal sensory and motor level(s). Further refinement of the trunk and abdomen testing paradigm that we created could include paraspinal EMG, and testing for artifacts from nearby structures. A clinical trunk score (68) and spirometry should be incorporated (15, 162, 163). Another approach to defining a level based on ICs might be to determine the level(s) above which IC spasms cannot be generated. Given that SSEPs did not substantially contribute to the research findings, future clinical trial protocols in complete injury may need to utilize sensory assessments evaluating contiguous dermatomes (12).

## Conclusions

This open-label, non-controlled, dose-escalation study cannot definitively establish a correlation between the positive electrophysiology findings and autologous Schwann cell transplantation. Electrophysiological testing revealed connections not appreciable from the clinical exam challenging the concept of equating the motor level to the NLI in the thoracoabdominal region. Although a primary outcome measure and minimally important clinical recovery are defined for pivotal efficacy studies (164), the expectation of observing such differences in early phase exploratory studies creates a risk for false negatives regarding the presence of therapeutic activity. This may lead to abandoning a therapeutic whose efficacy could be improved or potentiated. Voluntary EMG may be especially sensitive to reveal reorganized circuits that may involve several synapses and transmission delays. Based on these findings, we consider that the ISNCSCI sensory exam level may not be equivalent to the residual motor innervation of ICs and RAB muscles. However, the functional significance of the small amplitude potentials we observed is unclear.

Given that we detected considerable changes between 6 and 12 months as have others (28, 36), there is a rationale to study participants for more extended periods and determine if additional improvement occurs beyond 12 months.

For future studies, we recommend refinement of the trunk and abdomen testing paradigm that we created, the inclusion of paraspinal EMG, and testing for artifacts from nearby structures. A streamlined workflow is critical to permit this testing in a clinically feasible time frame.

## Data Availability Statement

The datasets generated for this study may be available in unidentified format through the corresponding author.

## Ethics Statement

The studies involving human participants were reviewed and approved by University of Miami, Institutional Review board. The patients/participants provided their written informed consent to participate in this study. The study was monitored by an independent data safety monitoring board.

## Author Contributions

AS performed electrophysiology assessments, analyzed data, and wrote and revised the manuscript. FB performed electrophysiology assessments, analyzed data. PS analyzed data. KA was the clinical study coordinator. AK was responsible for cellular preparations. ADL was a principal investigator for the trial. WDD was the study sponsor. JDG was also a principal investigator for the clinical trial and the electrophysiology group, performed electrophysiology assessments, analyzed data, and reviewed and revised the manuscript. All authors contributed to the article and approved the submitted version.

## Conflict of Interest

WDD, ADL, JDG, and AK have an ownership interest in Aceso Therapeutics, a spin-off company related to Schwann cell-derived therapeutics. This company had not been formed until after these studies were conducted. The remaining authors declare that the research was conducted in the absence of any commercial or financial relationships that could be construed as a potential conflict of interest. The reviewer, NW, declared a past co-authorship with one of the authors JDG to the handling editor.

## Supplementary Material

Figure S1 Thoracoabdominal recording sites. The electrode positions for the surface electrode intercostal recordings are shown. The NLI is indicated in purple. Needle electrodes were used to record from the rectus abdominus muscle groups between the lower sternum and pubic bone.

Figure S2 ISNCSCI NLI is indicated at the pre-transplant Baseline and 6 and 12 months post-transplantation.

Figure S3 TMS recruitment curves for C1/C2 versus CZ in a control subject with a readout in the TA muscle. The added effect of the Jendressick maneuver versus an estimated 10% maximum voluntary contraction is shown. We observed little difference in both the control and SCI subjects for Cz versus C1/C2 stimuli.

Figure S4 The median M wave stimulated APB is shown as well as the MEP obtained in the same setting for subject 006 at the 6-month study time point. The amplitudes used for calculation were the total peak-to-peak resulting in 8.8/14.18 = 0.62. The overall MEP/CMAP ratio for the APB from control sessions was relatively high at 0.58 ± 0.19 mV.

Figure S5 Voluntarily-evoked AH EMG at 2 months and 6 months, Subject 009-T6. Instructions are to initiate activity at the 4 second mark and stop at the 16 second mark. At 2 months, there is a delay in EMG onset and premature stopping. At 6 months, the ability to initiate and stop is much more convincing.

Figure S6 Detection of spasms during attempted voluntary movement. The left panel shows a single unit firing that generalizes and results in EMG activity that the subject could not stop at the cessation time point. The right panel shows EMG activity that was frequently observed during attempted tibialis anterior and abductor hallucis voluntary activation.

Figure S7 Upper panel. The thoracoabdominal breath hold EMG recordings contained EKG-like artifacts. Low pass filtering has been recommended to attenuate these. Lower panel. An effective filter in the Spike program was a low pass Chebyshev filter (Chavan et al., 2008) but this resulted in a large attenuation of the EMG signal.

Figure S8 Subject 006. Additional EMG channels T10 and AB1 with their respective RMS. Shown here are 4 caudally contiguous EMG channels that show the extent of EKG artifact in T9, an unusual possibly spastic artifact in T10, and spindles of activity in AB1 that likely represent a component of spasticity, although the subject was able to stop the signal.

Figure S9 Subject 002 brachial plexus injury and recovery. Right arm motor score recovery in the first year after aSC transplantation. This subject entered the study with a brachial plexus injury. A normal total motor score for one upper extremity per the ISNCSCI exam is 25. The x-axis is semi-logarithmic. An almost full recovery is observed, however at one year post-transplant the amplitudes of MEPs remained low. This suggests that a 5/5 muscle strength can occur while the evoked potentials remain depressed.
