# Supplementary figures and images for: Neurophysiological Changes in the First Year After Cell Transplantation in Sub-acute Complete Paraplegia
Source: Front Neurol. 2021 Jan 18;11:514181. doi: 10.3389/fneur.2020.514181 (PMC7848788; doi:10.3389/fneur.2020.514181)

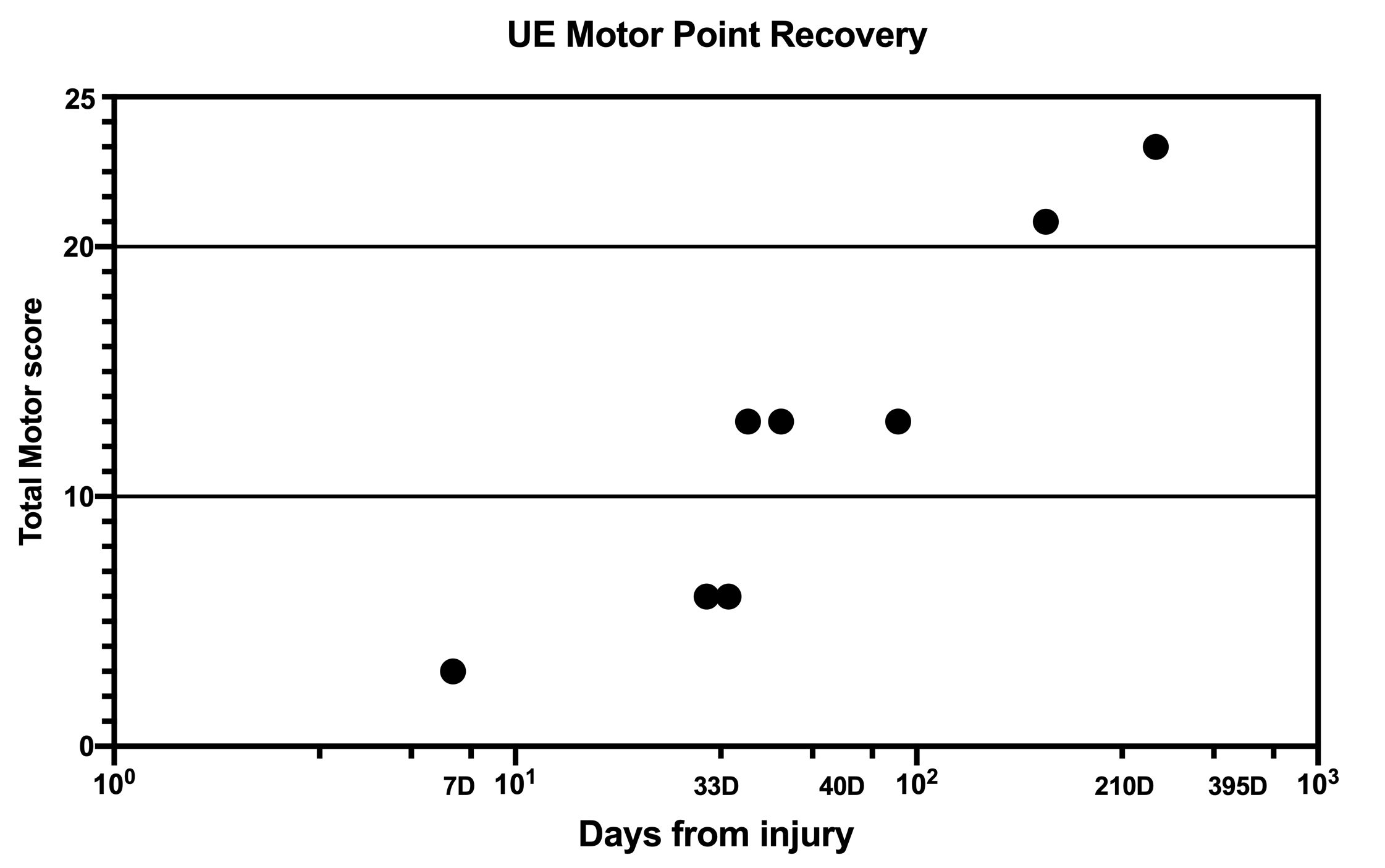

Supplement: Figure S1 — Thoracoabdominal recording sites. The electrode positions for the surface electrode intercostal recordings are shown. The NLI is indicated in purple. Needle electrodes were used to record from the rectus abdominus muscle groups between the lower sternum and pubic bone. [file Presentation_1.zip › Supplemental Figures 12.25.2020/Figure S9. Brachial plexus recovery semilog scale.tiff]

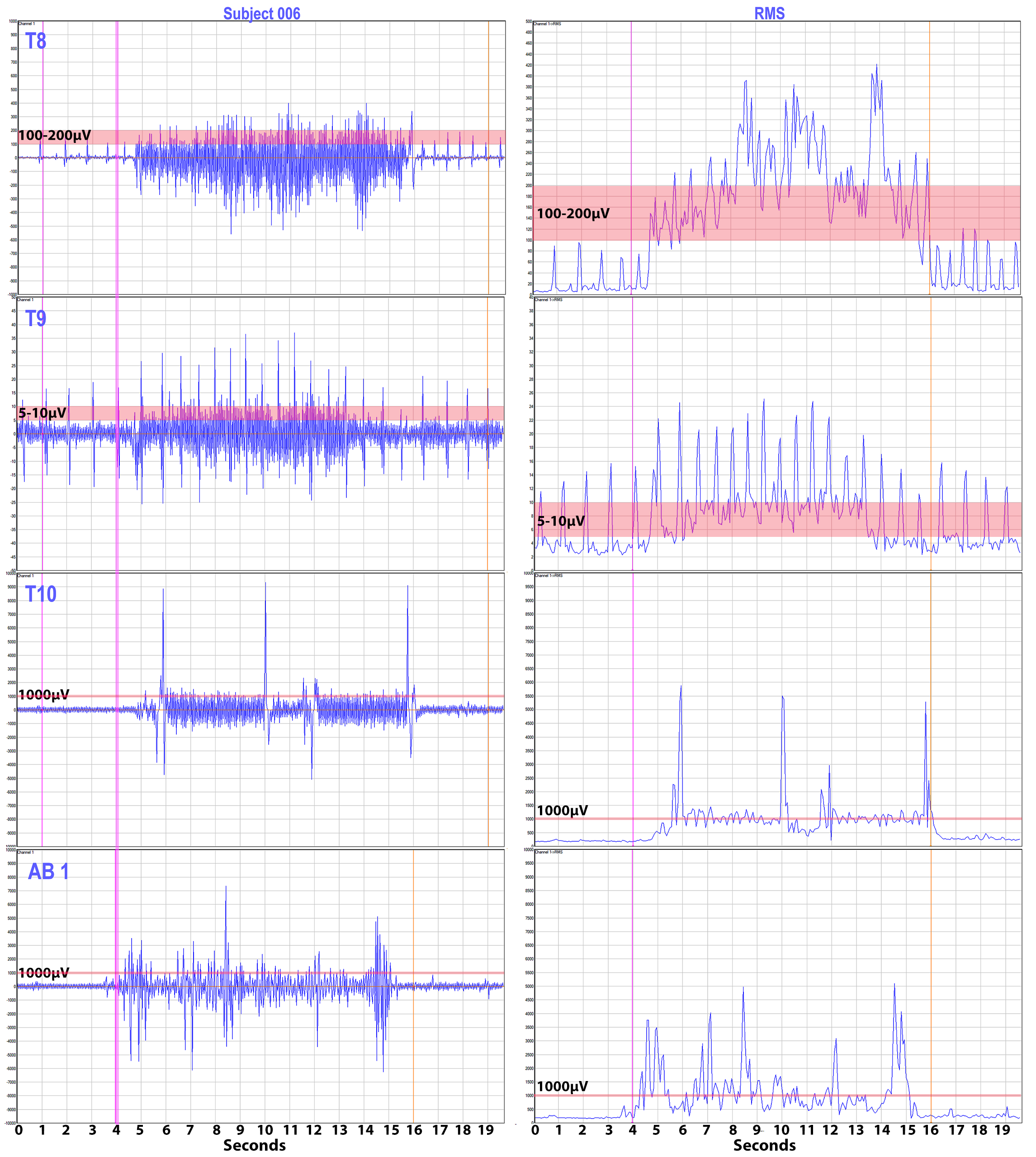

Supplement: Figure S1 — Thoracoabdominal recording sites. The electrode positions for the surface electrode intercostal recordings are shown. The NLI is indicated in purple. Needle electrodes were used to record from the rectus abdominus muscle groups between the lower sternum and pubic bone. [file Presentation_1.zip › Supplemental Figures 12.25.2020/Figure S8. Intercostal spastic activity.tif]

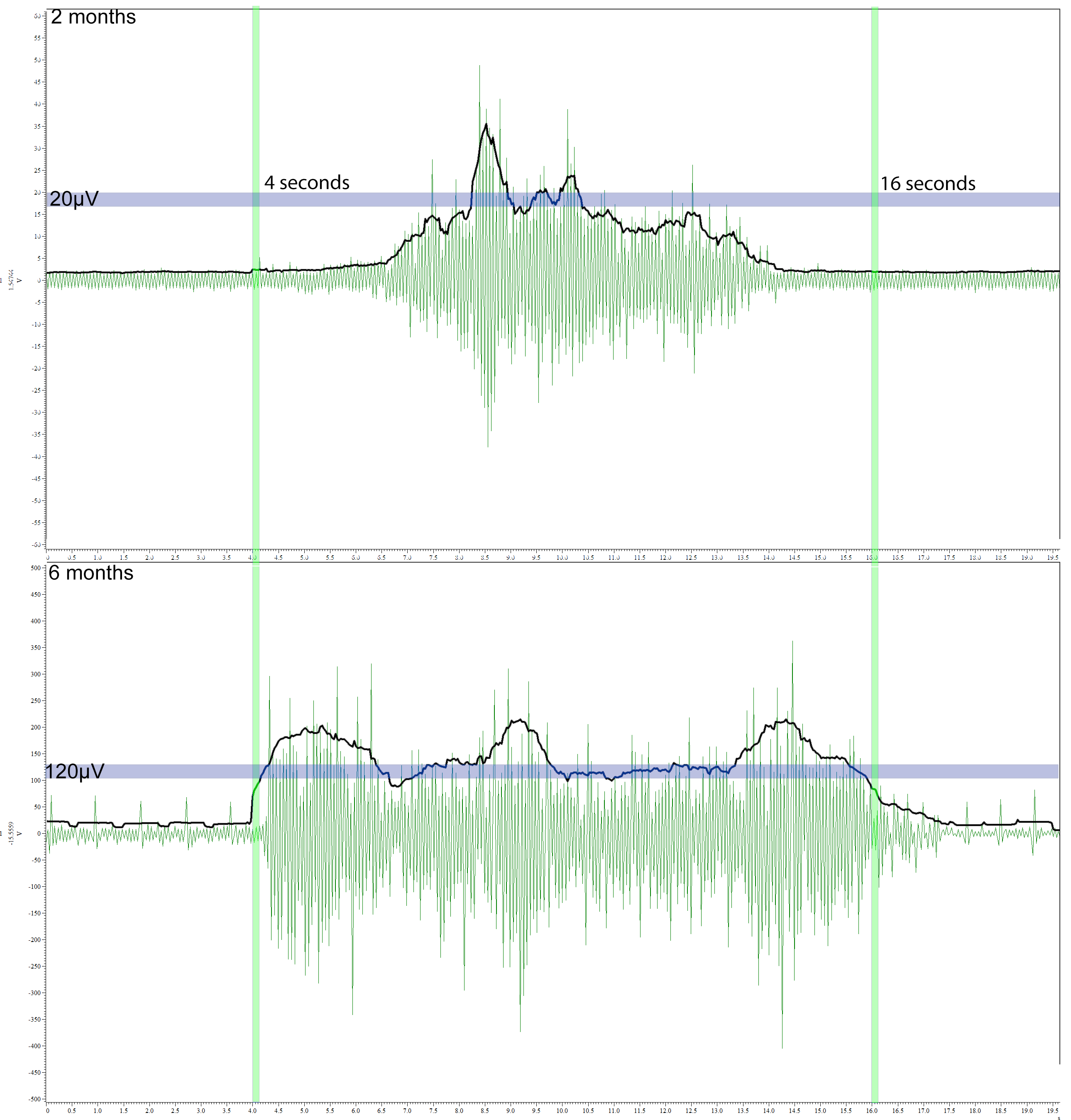

Supplement: Figure S1 — Thoracoabdominal recording sites. The electrode positions for the surface electrode intercostal recordings are shown. The NLI is indicated in purple. Needle electrodes were used to record from the rectus abdominus muscle groups between the lower sternum and pubic bone. [file Presentation_1.zip › Supplemental Figures 12.25.2020/Figure S5. Small amplitude voluntary EMG .tif]

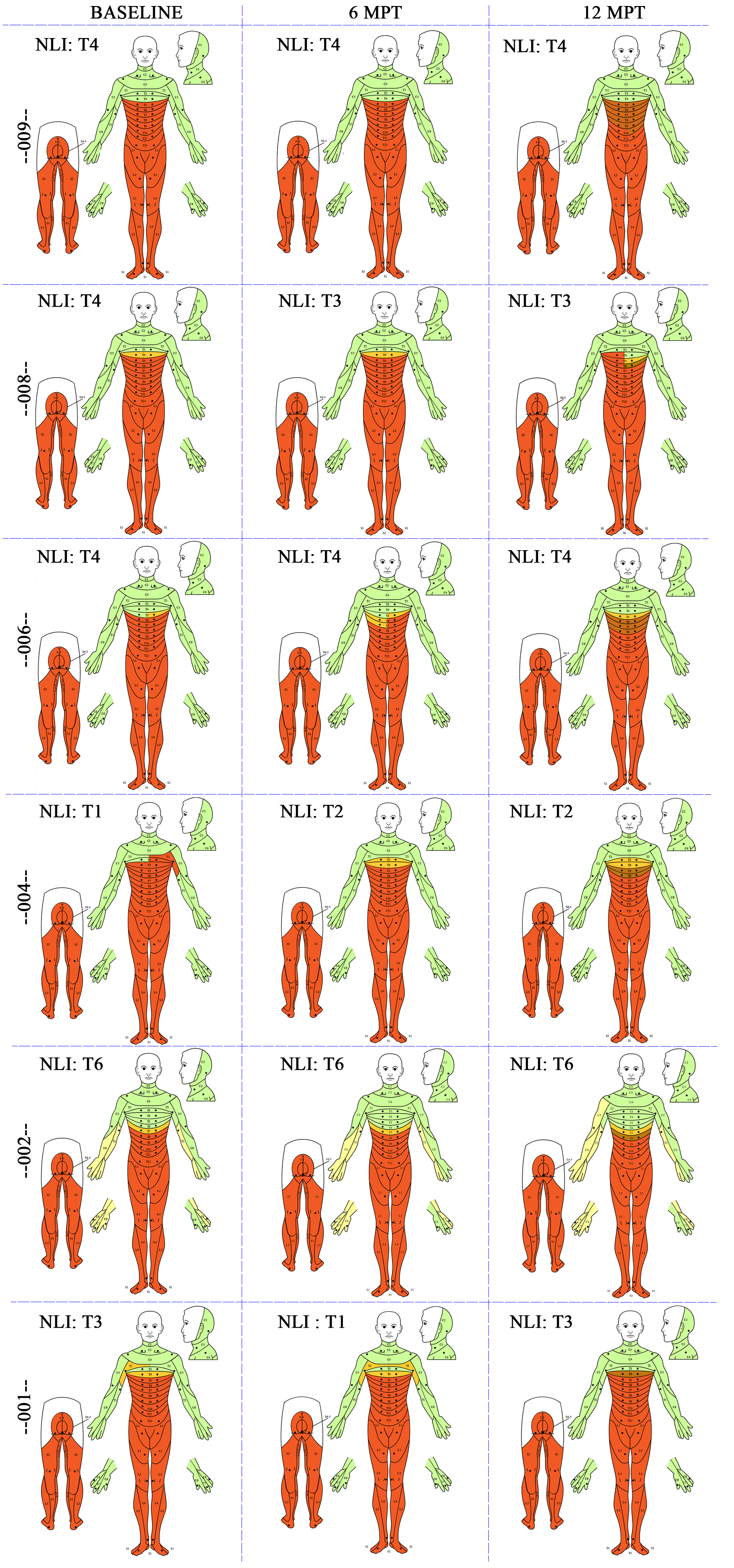

Supplement: Figure S1 — Thoracoabdominal recording sites. The electrode positions for the surface electrode intercostal recordings are shown. The NLI is indicated in purple. Needle electrodes were used to record from the rectus abdominus muscle groups between the lower sternum and pubic bone. [file Presentation_1.zip › Supplemental Figures 12.25.2020/Figure S2. ISNCSCI NLI.tif]

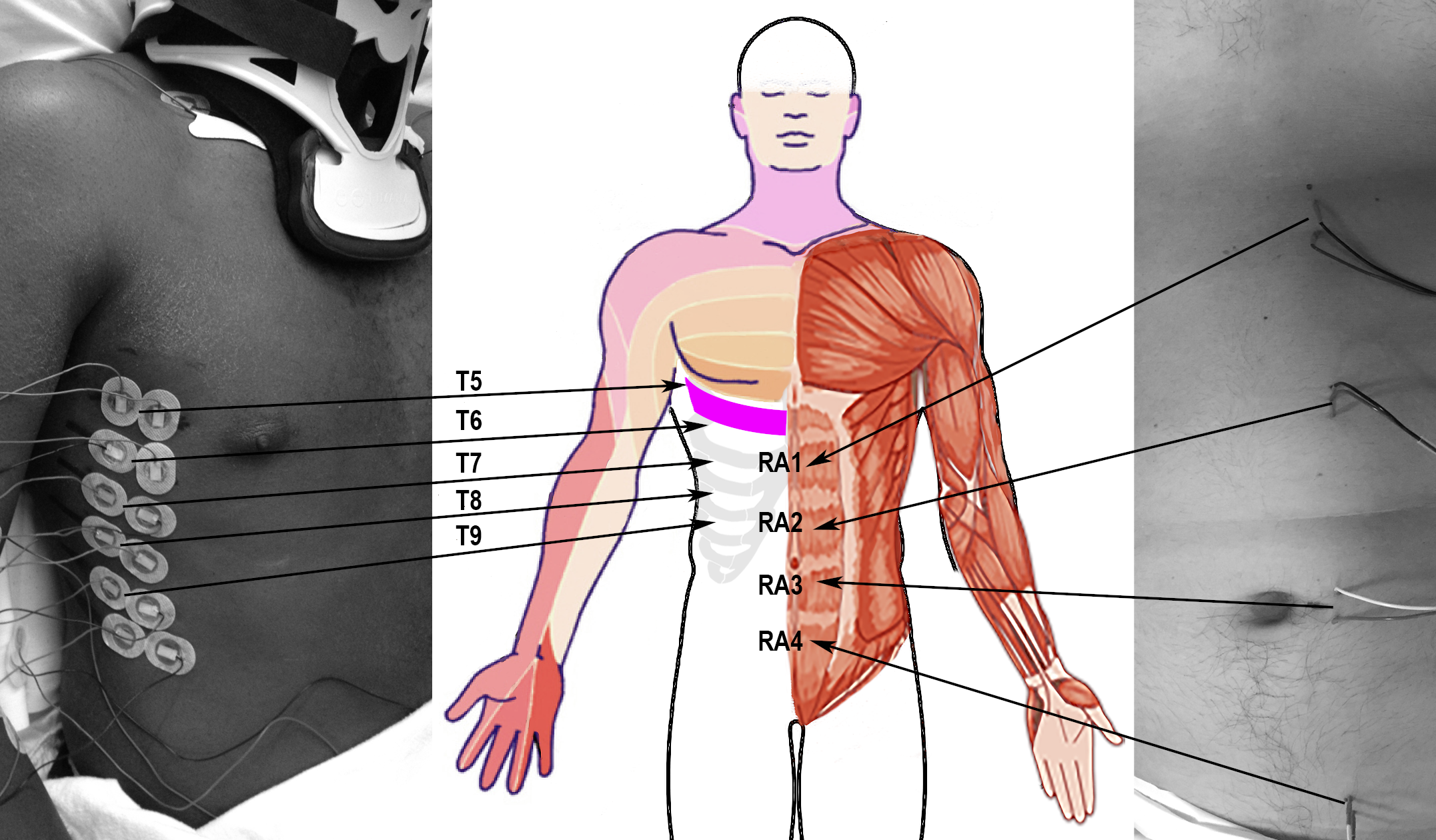

Supplement: Figure S1 — Thoracoabdominal recording sites. The electrode positions for the surface electrode intercostal recordings are shown. The NLI is indicated in purple. Needle electrodes were used to record from the rectus abdominus muscle groups between the lower sternum and pubic bone. [file Presentation_1.zip › Supplemental Figures 12.25.2020/Figure S1. Thoracoabdominal recording sites.tif]

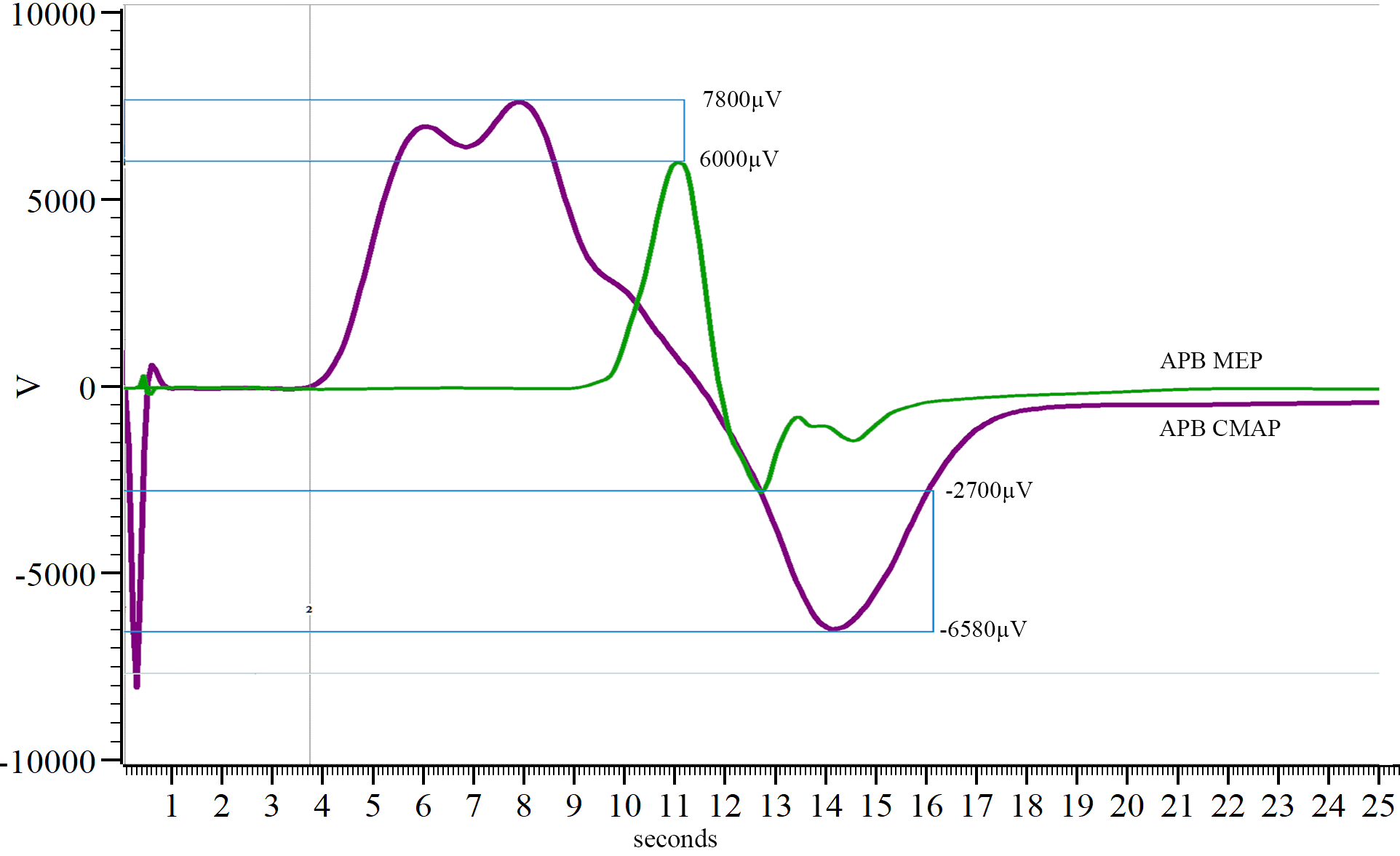

Supplement: Figure S1 — Thoracoabdominal recording sites. The electrode positions for the surface electrode intercostal recordings are shown. The NLI is indicated in purple. Needle electrodes were used to record from the rectus abdominus muscle groups between the lower sternum and pubic bone. [file Presentation_1.zip › Supplemental Figures 12.25.2020/Figure S4. M Wave example.tif]

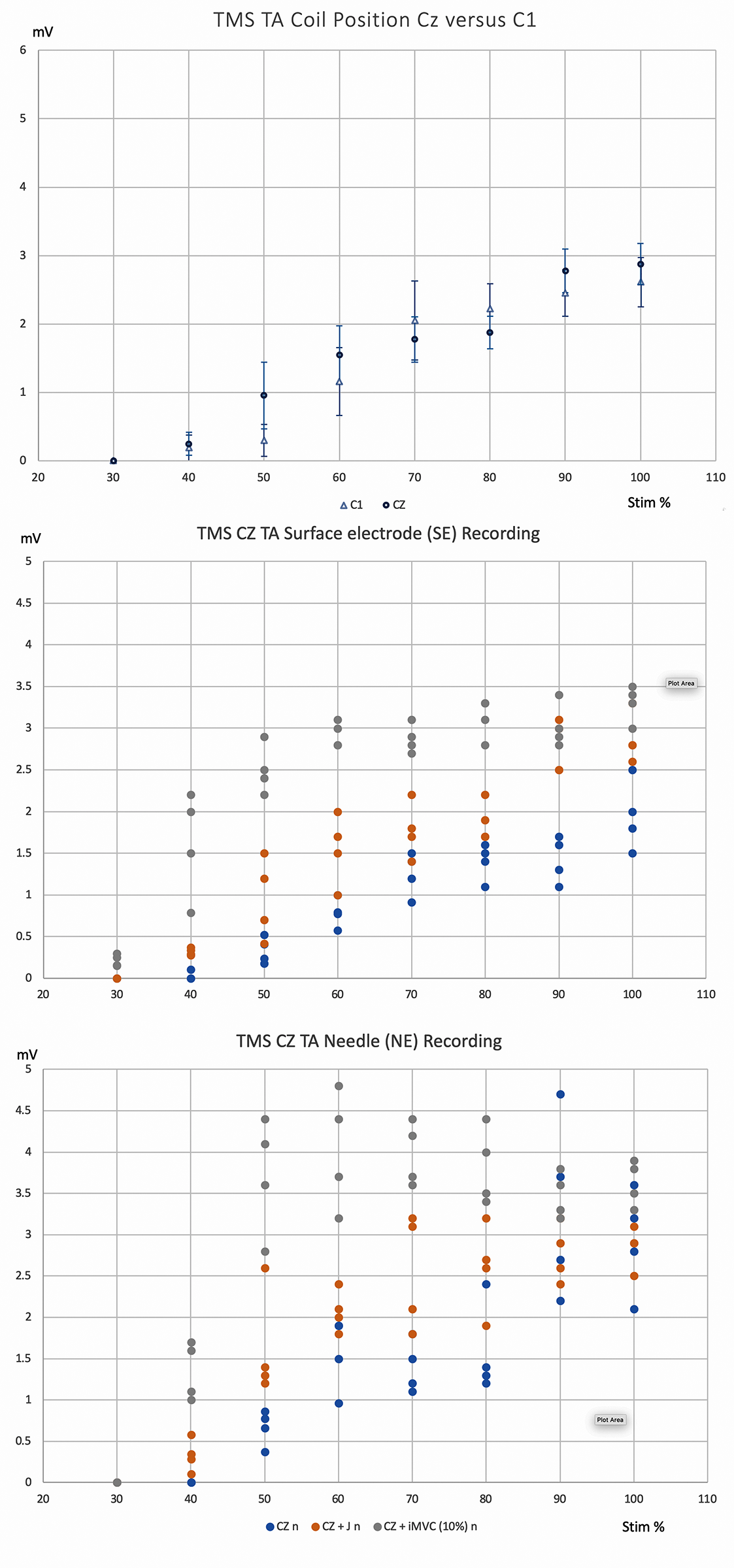

Supplement: Figure S1 — Thoracoabdominal recording sites. The electrode positions for the surface electrode intercostal recordings are shown. The NLI is indicated in purple. Needle electrodes were used to record from the rectus abdominus muscle groups between the lower sternum and pubic bone. [file Presentation_1.zip › Supplemental Figures 12.25.2020/Figure S3. TMS recruitment curves.tif]

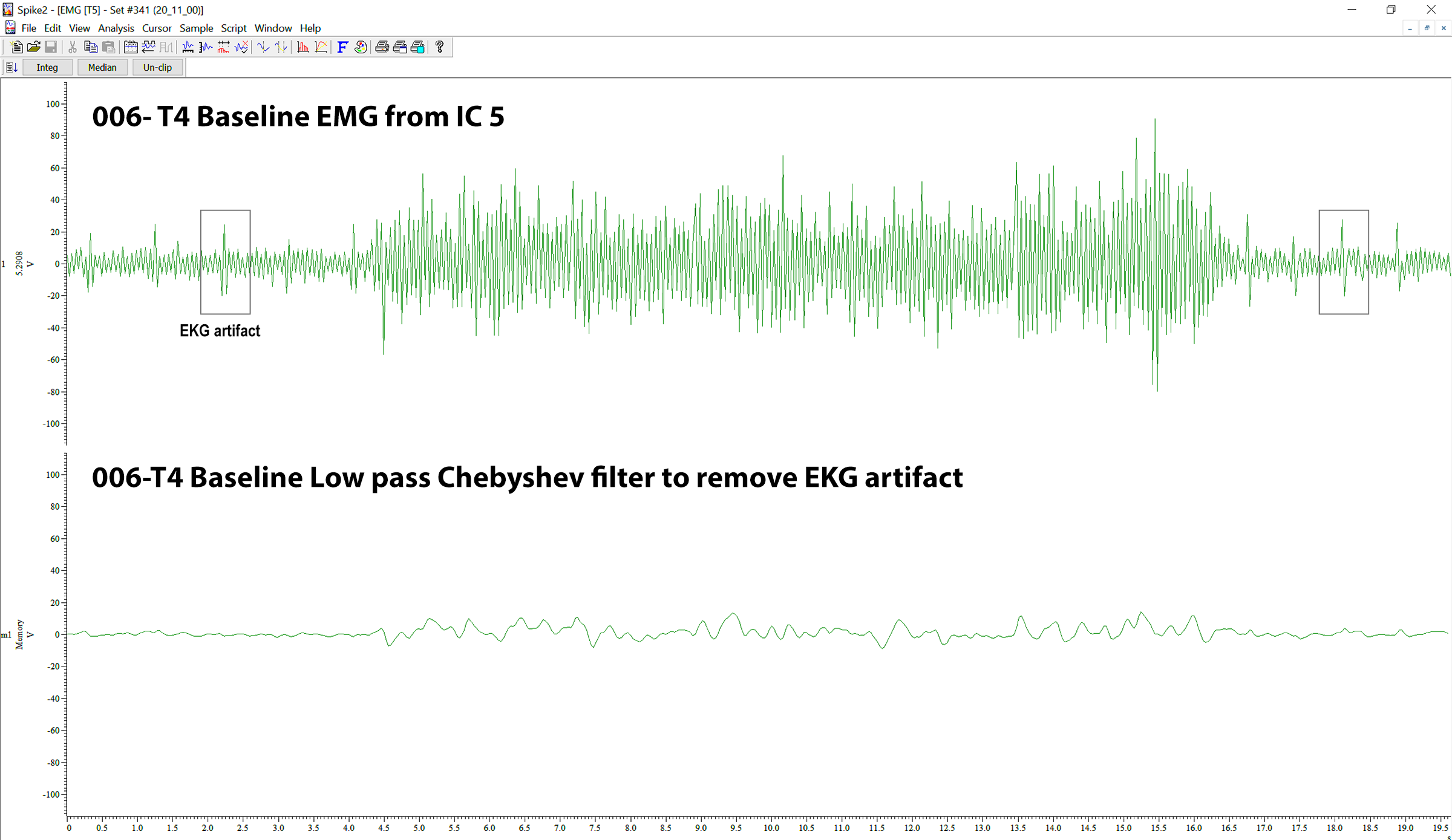

Supplement: Figure S1 — Thoracoabdominal recording sites. The electrode positions for the surface electrode intercostal recordings are shown. The NLI is indicated in purple. Needle electrodes were used to record from the rectus abdominus muscle groups between the lower sternum and pubic bone. [file Presentation_1.zip › Supplemental Figures 12.25.2020/Figure S7. Effect of low pass filter to remove EKG artifact.tif]

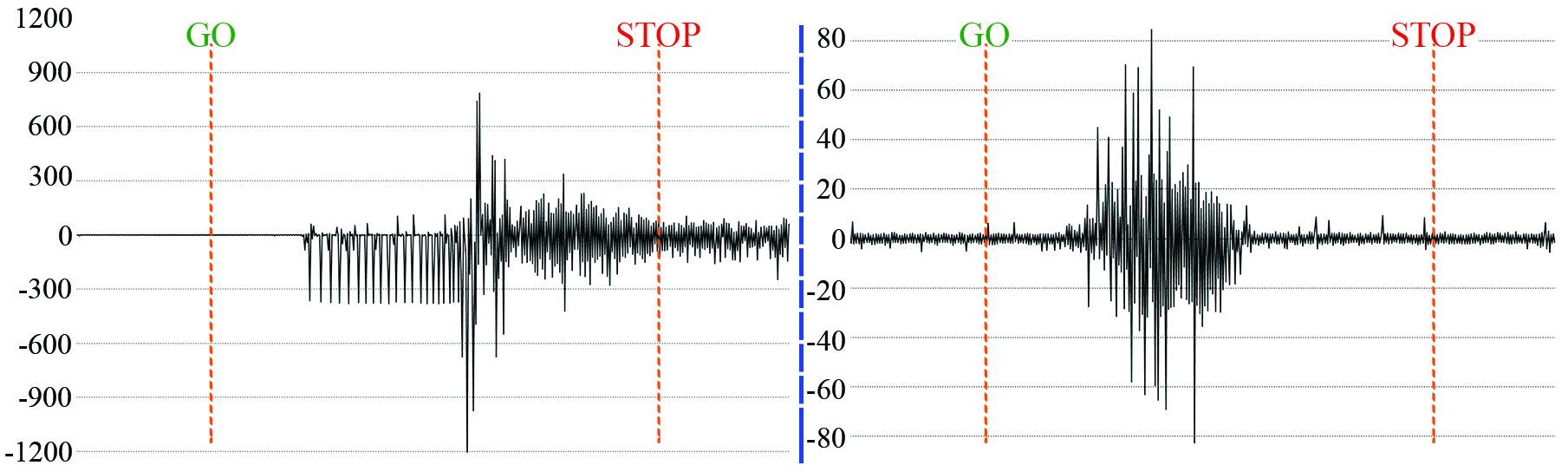

Supplement: Figure S1 — Thoracoabdominal recording sites. The electrode positions for the surface electrode intercostal recordings are shown. The NLI is indicated in purple. Needle electrodes were used to record from the rectus abdominus muscle groups between the lower sternum and pubic bone. [file Presentation_1.zip › Supplemental Figures 12.25.2020/Figure S6. Example of a spasm.tif]
